# Preliminary Efficacy of a Cognitive Behavioral Therapy Text Messaging Intervention Targeting Alcohol Use and Antiretroviral Therapy Adherence: A Randomized Clinical Trial

**DOI:** 10.1371/journal.pone.0229557

**Published:** 2020-03-12

**Authors:** Suzette Glasner, Helene Chokron Garneau, Alfonso Ang, Lara Ray, Alexandra Venegas, Richard Rawson, Seth Kalichman

**Affiliations:** 1 Department of Psychiatry and Biobehavioral Sciences, University of California Los Angeles, Los Angeles, CA, United States of America; 2 School of Nursing, University of California Los Angeles, Los Angeles, CA, United States of America; 3 Department of Psychology, University of California Los Angeles, Los Angeles, CA, United States of America; 4 Department of Psychiatry, Center for Behavior and Health, University of Vermont, Burlington, Vermont, United States of America; 5 Institute for Collaboration on Health, Intervention, and Policy, University of Connecticut, Mansfield, Connecticut, United States of America; Brown University, UNITED STATES

## Abstract

To date, no studies have reported the use of text messaging to deliver cognitive behavioral therapy (CBT) to alcohol users with HIV. We developed and evaluated a text messaging-based program to deliver CBT targeting both alcohol use and adherence to antiretroviral (ART) medications for adults with alcohol use disorders and comorbid HIV. Participants were randomly assigned to receive either a 12-week CBT-based text messaging intervention (ALC-TXT-CBT) targeting ART adherence and alcohol use (n = 18); or an informational pamphlet (INFO) comprising educational content focused on alcohol use and medication adherence (n = 17). ART adherence and alcohol use were compared for those in ALC-TXT-CBT to INFO across baseline and 12-week (i.e., treatment-end) assessments using mixed model regression analyses and paired t-tests. Relative to those in INFO, ALC-TXT-CBT participants evidenced increases in ART adherence, measured by phone-based unannounced pill counts (β = .16, p = .04), and biochemically by viral load (t = -2.0, p = .04) at treatment-end. ALC-TXT-CBT participation was also associated with reductions in heavy drinking at treatment-end (t = 2.7, p = 0.02), while overall alcohol use frequency did not differ significantly between groups (t = 1.5, p = 0.07). Findings demonstrated promising preliminary evidence for the efficacy of ALC-TXT-CBT in improving medication adherence and reducing heavy alcohol use among individuals with alcohol use disorders and comorbid HIV infection.

**Trial registration:**

This trial was registered with ClinicalTrials.gov, NCT02603471.

## Introduction

Antiretroviral therapy (ART) is a highly effective treatment for reducing human immunodeficiency virus (HIV) replication among individuals with HIV infection to undetectable levels [1]. Owing largely to the introduction of ART regimens, approximately half of those with HIV in the U.S. are successfully achieving viral suppression [2]. However, the clinical benefits of ART are dependent on high levels of ART adherence; likewise, suboptimal adherence is associated with viral resistance to treatment and an array of negative consequences including rebounding of HIV RNA levels, sometimes to above baseline levels [3–8]. Alcohol use is associated with greater disease burden and mortality among those who become HIV-infected, due, in large part, to the inverse relationship between alcohol consumption and adherence to HIV treatment [5,6].

There is ample evidence suggesting that HIV-infected alcohol users are less likely to access HIV treatment and that once treatment is initiated, their risk of non-adherence is high, relative to former and non-users [9]. Heavy alcohol use has been shown to have a marked impact on adherence by diminishing one’s capacity to plan for or remember dosing requirements [10]. Additionally, recent work by Kalichman and colleagues suggests that poor adherence among alcohol users may be intentional, resulting in part from alcohol-ART interactive toxicity beliefs [11,12]. Likewise, according to a meta-analysis, alcohol users are 50–60% as likely to adhere to ART, relative to those who abstain, or drink relatively small quantities of alcohol [13]. Collectively, these data suggest that interventions targeting ART adherence are needed to optimize the treatment outcomes of alcohol users who are living with HIV. To this end, intervention components that may improve outcomes include: (1) evidence-based therapeutic skills training to facilitate reductions in or abstinence from alcohol use; (2) education about the importance of adherence to ART along with risks associated with poor or inconsistent adherence; and (3) behavioral skills training to bolster adherence. In the present study, we developed and evaluated, in a pilot randomized clinical trial, a technology-assisted behavioral intervention that addresses all 3 of these potential need areas, delivered via text messaging.

Text messaging has shown promise as an approach to improving health outcomes among both populations with HIV [13–17] and those with problematic alcohol use [18]. In a study of young adult alcohol users, an interactive text messaging intervention targeting binge drinking was associated with reductions in alcohol consumption and alcohol related injury prevalence at 6 month follow-up, relative to controls [19]. Likewise, a systematic review that examined outcomes of mobile health interventions targeting unhealthy alcohol use reported that 11 out of 18 studies demonstrated benefits of these approaches on drinking outcomes including alcohol use frequency, heavy drinking days, and binge drinking [20]. Text messaging interventions for people living with HIV have been the focus of various systematic reviews and meta-analyses, confirming that these approaches improve various parameters of HIV care, particularly ART adherence [21–25]. Nevertheless, to our knowledge, integrated interventions using text messaging to target both alcohol use and ART adherence concurrently have not been the focus of prior investigations. Furthermore, while extant studies have relied primarily on text messaging reminder systems [21], a relatively small body of literature, largely from the ecological momentary assessment (EMA) area, has examined the potential efficacy of evidence-based, theory driven therapeutic strategies delivered via text messaging [26]The approach, often referred to as ecological momentary intervention (EMI), of incorporating EMA strategies into behavioral interventions, can employ adjunctive strategies to behavioral treatments that include: (a) real time self-monitoring of symptoms and behaviors [27]; (b) in vivo skills training [28], and (c) delivery of individualized feedback, particularly when an individual enters a risky situation [29]. The present study incorporates elements of the EMI model in the development of a CBT intervention targeting alcohol use and ART adherence.

Cognitive behavioral therapy (CBT)- based ART adherence counseling [4,30,31] is effective in bolstering adherence among substance users living with HIV [4,30,31]. Moreover, the therapeutic effects of CBT as an intervention strategy for problematic alcohol use are robust and have been well established among those with alcohol use disorders [32]. Although CBT targeting alcohol use has been effectively transported to technology-assisted, computerized delivery mediums including CBT4CBT [33] and Self Help for Alcohol and other Drug Use and Depression (SHADE) [33] with demonstrated efficacy in improving alcohol use outcomes, similar efforts to deliver CBT using text messaging have not been documented.

Text messaging is a particularly appealing approach to disease management for individuals with HIV and substance use comorbidity because it is a highly scalable, low cost, user-friendly, ubiquitous and flexible approach. Our team recently developed and evaluated a CBT based intervention delivered using text messaging for individuals with drug use disorders (i.e., opioid and stimulant users) living with HIV [34]. Several features of the text messaging intervention were designed to retain the core elements of face-to-face CBT: first, the intervention involved a face-to-face session with a clinician in which CBT concepts were introduced and individualized to each participant’s skills training needs. For example, adherence focused CBT concepts introduced in the face-to-face session included problem solving around barriers to adherence (e.g., transportation problems that led to missed clinic visits), identifying maladaptive thoughts underlying poor adherence and challenging them, and introducing behavioral strategies (e.g., cue control) to mitigate forgetting to take medications. Second, the texting intervention facilitated skills training by reinforcing these concepts with text messages in which participants were reminded of the themes discussed with the clinician, including the concrete behaviorally themed plans that were made to overcome barriers to adherence. Third, participants were encouraged via personalized text messages to implement the plans that were made in the face-to-face session, and to evaluate whether the plans were effective. Likewise, to promote relapse prevention skills, tailored and bidirectional text messages were delivered to remind participants of their individual motives for changing their substance use, as well as assisting them in developing a personalized relapse prevention plan using CBT skills and concepts that were introduced via text messages over the course of the intervention.

Preliminary outcomes demonstrate promise of this intervention, which effectively increased ART adherence and reduced drug use [35]. In the present study, with user input, we developed [36] and tested, in a pilot RCT, an integrated, CBT-based, bidirectional text messaging intervention, targeting alcohol use and ART adherence in a population of adults with HIV-alcohol use disorder comorbidity. Here, we report the preliminary efficacy of the ALC-TXT-CBT intervention in reducing alcohol use and improving ART adherence.

## Methods

### Participants

Eligibility criteria for the study were as follows: 18 years of age or older, HIV positive and currently taking ART prescribed within the past 30 days (as confirmed by medical records from their HIV care provider), mean ART adherence of 90% or less, as determined by the unannounced pill count procedure (described below), current DSM-V diagnosis of alcohol use disorder, and owning a cellular phone with text messaging capabilities. Potential participants were excluded from the study if they: lacked proficiency in English, were currently homeless, according to self-report (unless residing in a recovery home for which contact information could be provided), were dependent on an illicit substance for which medical detoxification was imminently needed, or presented clinically significant psychiatric symptoms as assessed by MINI International Neuropsychiatric Interview, such as psychosis, acute mania, or suicide risk that would require immediate treatment or make study compliance difficult.

### Procedure

Participants receiving treatment as usual for HIV infection were randomly assigned to receive either the text-messaging CBT intervention (ALC-TXT-CBT) or an informational pamphlet concerning HIV and alcohol use (INFO).

Recruitment for the study was conducted from June 2014 to May 2016, when the funding period terminated. Participants were recruited using advertising, word of mouth, study announcement flyers posted in treatment programs and community locations, including infectious disease clinics, referrals from local substance abuse treatment and outreach programs, primary care providers, local mental health centers, and crisis clinics. All recruitment materials referred interested persons to clinic phone numbers, which were answered by trained research staff who provided the caller with information about the study. Interested individuals were given a detailed description of the study and were pre-screened by phone prior to an initial interview to begin the informed consent process. Those who were determined to be eligible based upon the pre-screening provided written informed consent, at which time the baseline assessment procedures, including administration of questionnaires concerning substance use, as well as a phone-based unannounced pill count procedure to evaluate ART adherence, were initiated. Following informed consent, participants were randomly assigned to one of the two study conditions (ALC-TXT-CBT or INFO). The computer-generated randomization list was prepared by the trial statistician. Randomization was web-based and was conducted by a trained research assistant. Neither the research staff nor participants were masked to the treatment allocation.

The target sample size (N = 50) was guided by appropriateness for pilot study objectives [37,38], allowing for detection of an effect size of about d = .7 in differential change in target outcomes from baseline to follow-up, assuming a moderate correlation of 0.50 over time and allowing attrition of up to 20% [39]. While we were able to achieve 70% of this target sample size, as reported further below, group differences were nonethelesss detected on key outcomes.

**Fig 1:**
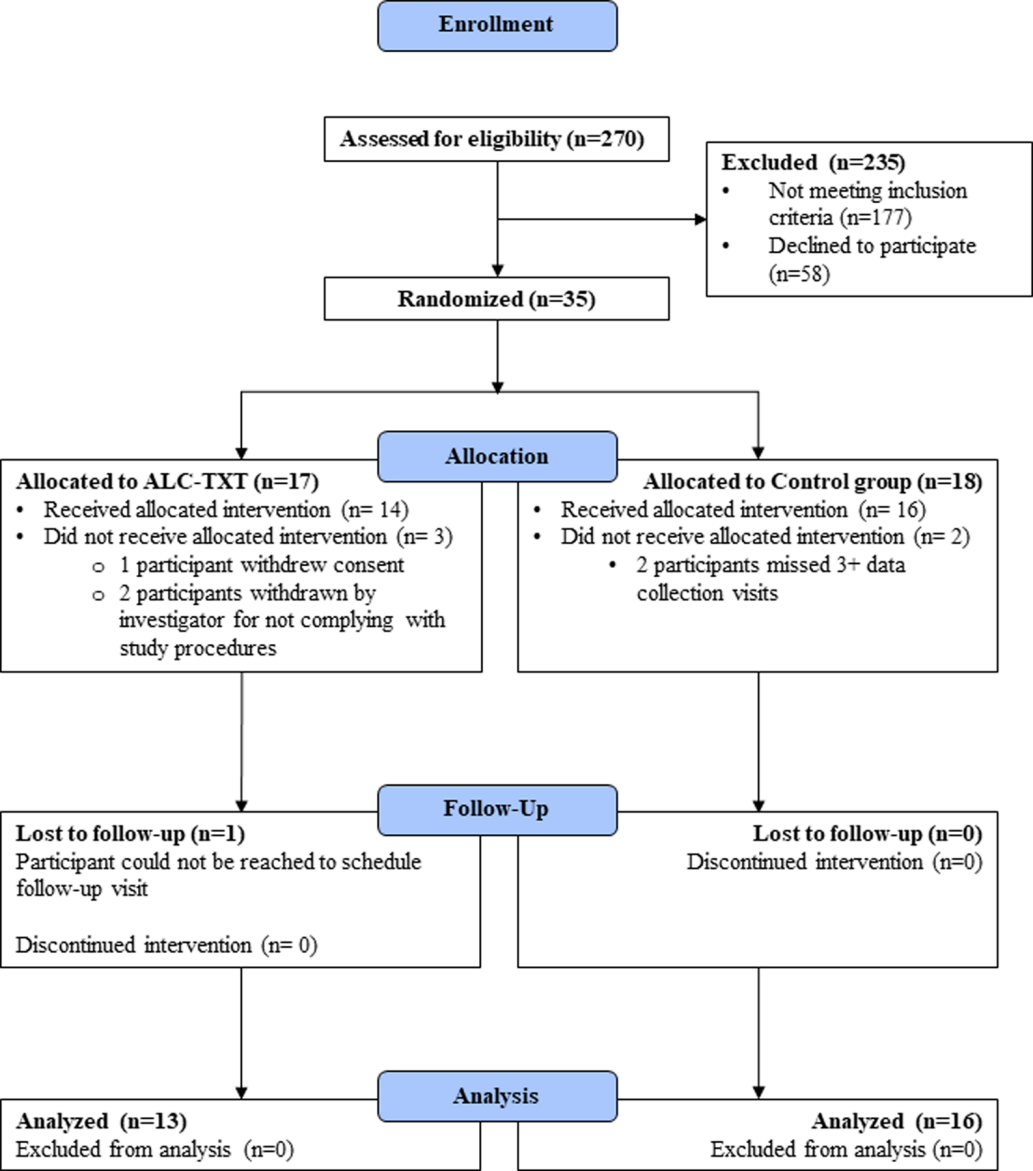
CONSORT flow diagram.

The trial was conducted and reported in accordance with the CONSORT guidelines [40]. Fig 1 (Fig 1) depicts the study participant flow. Face-to-face and phone-based data collection assessments were conducted at baseline, weeks 4, 8, and 12 (i.e., treatment end). The majority of the assessments were conducted using a web-based data entry system. Participants were compensated using gift cards as follows: $40 for the baseline assessment, $20 for the 4-, 8- and 12-week follow-up assessment, and $20 for each phone-based pill count procedure. At the baseline visit, demographic, clinical, and risk behavior data were collected. Participants were compensated $20 both at baseline and treatment-end for providing their most recent laboratory report from their HIV treatment provider indicating viral load data. There were no significant group differences in follow-up rates between the two study conditions. All study procedures were approved by the UCLA Institutional Review Board. The trial was registered at ClinicalTrials.gov, NCT02603471.

### ALC-TXT-CBT intervention

The ALC-TXT-CBT intervention was developed in a formative process comprising a series of focus groups to acquire user input regarding intervention content, coupled with community partner and CBT expert groups in which content was reviewed and finalized [36]. The ALC-TXT-CBT intervention comprised a single CBT-based face-to-face counseling session delivered by a master’s-level clinician, which was adapted from LifeSteps [4] an empirically based CBT approach to facilitating medication adherence, followed by 12 weeks of daily text messages. Text messages included medication reminders, delivered at one or more times of day specified by the participant, plus the participants’ selected frequency of additional messages (either 2 or 3 to be delivered daily) on the topics of addiction recovery, relapse prevention, and associated risk behaviors. Approximately 50% of the message content, apart from medication reminders, comprised CBT skills training targeting alcohol relapse prevention, with the remaining half addressing adherence benefits and risks of nonadherence (25%), and behavioral skills training germane to ART adherence (25%).

The clinician-delivered CBT session was designed to identify intervention “tailoring variables,” or clinical/behavioral data that provided the basis for individualizing the text messaging content, including: (1) the top 3 barriers to ART adherence, with corresponding plans for coping in a manner that would facilitate taking medications as prescribed, (2) the specific times of day at which to deliver medication reminders, and (3) motives for stopping or maintaining changes in substance use (i.e., we asked participants to list motivating reasons for quitting, or maintaining changes in substance use to be incorporated in tailored messages that would be sent in response to their use of the “CRAVE” function).. Participants’ first names were also programmed for the purposes of personalizing messaging content.

Content included both educational (i.e., unidirectional) and interactive (bidirectional) components, to model the balance between psychoeducation and behavioral counseling in clinician-delivered CBT. Intervention content is described in detail elsewhere [34]. In brief, throughout the 12-week intervention, topics changed on a weekly basis, with thematic emphasis rotating between the following coping skills: (1) Behavioral strategies (e.g., scheduling and engaging in pleasurable activities) to facilitate recovery; (2) Managing difficult emotions; (3) Stress management techniques; (4) Identifying common relapse triggers; (5) Relapse analysis practice; (6) Goal-setting, including development of goals that are incompatible with alcohol use; (7) Family and social support for recovery; (8) The impact of alcoholism and the recovery process on important relationships; (9) Behavioral lifestyle changes that support recovery; (10) Decisions about “other substance use” (e.g., marijuana); (11) Motivation for sustained recovery; and (12) Individualized relapse prevention planning.

#### ART adherence educational content

Informed by the Cognitive Social Health Information Processing model [41], benefits of adherence and risks of nonadherence were addressed via psychoeducational messages regarding routes of HIV transmission, medical consequences of ART nonadherence and health benefits of adherence, the impact of treatment on transmission and overall well-being, alcohol use and sexual risk behaviors, and ways to prevent transmission.

#### ART adherence behavioral skills training

Messages targeting ART adherence were based upon LifeSteps [4], a CBT program comprising education, problem solving, and strategies to promote medication adherence skills. Based on the top three barriers to adherence identified during the face-to-face clinician delivered CBT session, content was individualized to include education and reminders about plans developed in the session to utilize specific skills to optimize adherence, including: scheduling, cue control strategies, challenging maladaptive thoughts about adherence, and improving communication with medical providers [30].

#### Text “CRAVE”

Participants had the option to send a CRAVE text in real time when experiencing an urge to use alcohol. The CRAVE function generated immediate, personalized feedback based on the face-to-face session [42]to model social support as a means of coping, a key element of CBT for relapse prevention.

### Treatment as usual intervention content

As an eligibility criterion was currently taking ART, all participants in both conditions were receiving usual HIV care, which comprised pharmacological management by a physician in an infectious diseases clinic setting. Generally, participants were scheduled for monthly visits with their physician and quarterly assessment of viral load.

#### Clinician involvement

Apart from the face-to-face CBT session provided at the beginning of the ALC-TXT-CBT condition, participants in this condition also had the opportunity for contact with a CBT clinician in the following contexts: (1) The text CALL function: In a subset of tailored text messages, participants were prompted to text the word CALL if they required further instruction about how to implement a particular coping skill that they had planned to use to facilitate ART adherence. If a text CALL response was received, the clinician called the participant back and provided counseling to assist with the specific skill for which guidance was needed. (2) The text LIFELINE function: Participants were able to use this function a total of three times during the 12-week intervention. Participants could text the word LIFELINE if they needed to speak to a live counselor during business hours. For both text CALL and text LIFELINE functions, participants received an automated response to remind them of the hours during which the counselor was available and were instructed to call 911 or go to an emergency room for any urgent matters beyond these hours or that required an immediate response. If the text was received during business hours, the clinician responded by calling the participant on the same day; if after hours the participant was contacted by phone on the following business day.

### Measures

#### Alcohol and other drug use

The Addiction Severity Index (ASI) was used to assess alcohol and other drug use frequency as well as heavy alcohol use in the 30 days prior at baseline and treatment end [43]. The ASI was used in conjunction with a calendar to identify the days on which alcohol use occurred, and participants were probed as to whether the individual drank to intoxication on each of the days in which alcohol use was reported.

#### ART adherence

ART adherence was assessed using both unannounced phone-based pill counts (UPCs) at baseline and monthly through treatment-end, and viral load data as a biochemical indicator at baseline and treatment-end. Both home- and phone-based UPCs are reliable and valid measures of ART adherence, yielding comparable data to electronic medication monitoring [44,45], and significant correspondence with plasma viral load [46]. At the initial research visit, participants were trained by an intake assessor in procedures that they would be asked to follow by phone when they were contacted for UPCs. Participants were trained to count their medications using the following steps after answering the telephone: (1) bring all medications that are in the home to a comfortable flat surface near the telephone, including closed bottles, pocketed doses, and pill boxes; (2) sort medications into clusters; (3) select a medication and tell the pill counter the prescription numbers, refill dates, number of refills remaining, and dispensed quantities; (4) report to the pill counter lost or gained pills since their previous count and whether the drug was taken that day; (5) count pills using pharmacist tray and cup provided by the study; if using a pillbox, open each compartment to count the pills without removing them from containers; (6) repeat procedure to double count all pills.

The first UPC was conducted within 1 week of the training, with subsequent monthly UPCs at unannounced times during the intervention and at treatment-end (i.e., week 12). All UPCs were conducted for each ART medication currently taken by the participant. Pharmacy information from pill bottles was also collected to verify the number of pills dispensed between calls. A medication adherence score was then calculated for each medication as the ratio of pills counted relative to pills prescribed, taking into account the number of pills dispensed. A data form was used to tabulate all pills counted, which included all of the data needed to calculate adherence, including dates that medications may have been stopped and started between pill counts. To calculate adherence scores at each timepoint, the difference between pills counted at the two times was divided by the pills prescribed, taking into account the number of pills dispensed, pills lost, gained, and taken that day. Stopped medications were adjusted for number of days between the previous pill count and the stop date. Medication refill information, specifically the filled dates and remaining number of refills were used to verify the accuracy of medications dispensed over the course of the pill counts.

Changes in adherence, averaged over medications in the ART regimen, was examined as the primary outcome [47]. Viral load served as a biological indicator of ART adherence, Consistent with the typical frequency with which viral load is assessed in clinical settings, data concerning viral load were collected at baseline and treatment end. Viral load data were not collected in the research setting; rather, participants either provided a copy of their latest viral load results, or signed a medical release of information allowing the research team to obtain their viral load results directly from their medical provider. Dates of baseline versus week 12 laboratory reports were compared as a quality control measure, to ensure that those provided at each of the timepoints were not duplicated.

### Statistical analysis

Primary outcome measures included ART adherence scores (based on unannounced pill count procedures conducted monthly during the 12-week intervention phase and at treatment-end), viral load (collected at baseline and at treatment-end), and alcohol use (i.e., overall frequency and heavy alcohol use frequency) in the 30 days prior to baseline and treatment-end. Viral load data were log transformed for purposes of data analysis. To evaluate between-subjects differences at each individual timepoint, bivariate analyses using t-tests were first conducted. This was followed by longitudinal mixed model analyses [48,49], to examine between-subjects differences in ART adherence for those in ALC-TXT-CBT relative to INFO across time, from baseline to the week 12 (i.e., treatment-end) assessments. Paired t-tests were used to examine pre- to post-treatment differences in alcohol use as a function of group assignment. Though there were no missing viral load data at baseline, because there were 8 missing viral load data at treatment-end, we handled these data using multiple imputation, with n = 10 imputations to fill in the missing values for each incomplete case. Alpha was set at .05 for all statistical analyses. All analyses were conducted using SAS 9.4 and STATA 15 software.

## Results

### Participant characteristics and ALC-TXT-CBT utilization

As shown in Table 1, participants were, on average, 50.2 (SD = 10.2) years of age. Mean years of educational attainment was 12.4 (SD = 3.1). The sample was predominantly male (84%), never married (53%), and African American (60%). On average, participants reported alcohol use on 13.1 of the past 30 days (SD = 10.5) at baseline, and alcohol use to intoxication on 6.7 of the past 30 days (SD = 8.6). Other drug use was not particularly prominent in this sample, with the most heavily used substances including cannabis, with an average in the overall sample of 7.9 days using out of the past 30 (SD = 11.9) and cocaine, with an average of 1.8 days using out of the past 30 (SD = 5.7). The average number of days of reported opioid or methamphetamine use was less than 1. There was no significant difference between age, ethnicity, educational attainment, gender distribution, employment status, and marital status between the two conditions. There also were no significant baseline differences between the study conditions in ART adherence, alcohol use, other drug use, and viral load.

**Table 1:** Sample characteristics.

	Sample N = 35	TXT-CBT N = 17	Control N = 18
Age, M (SD)	50.2 (10.2)	47.6 (11.5)	52.7 (6.1)
Gender, N (%)			
Male	30 (85.7)	16 (53.3)	14 (46.7)
Female	5 (14.3)	1 (20.0)	4 (80.0)
Yrs Education, M (SD)	12.4 (3.1)	11.8 (1.8)	12.9 (3.9)
Race, N (%)			
White	5 (14.3)	1 (20.0)	4 (80.0)
African-American	21 (60.0)	11 (55.6)	10 (55.6)
Hispanic	9 (25.7)	5 (55.6)	4 (45.4)
Employment, N (%)			
Full time	6 (17.1)	2 (33.3)	4 (57.1)
Part time	4 (11.4)	2 (50.0)	2 (50.0)
Retired/disabled	20 (57.2)	11 (55.0)	9 (45.0)
Unemployed	5 (14.3)	2 (40.0)	3 (60.0)
Marital status, N (%)			
Legally married/Living with partner	4 11.8)	3 (75.0)	1 (25.0)
Separated/Divorced/Widowed	12 (35.3)	6 (50.0)	6 (50.0)
Never married	18 (52.9)	8 (44.4)	10 (55.6)
Alcohol Use (past 30 days)			
Days of alcohol use, M (sd)	13.1 (10.5)	11.8 (9.7)	14.3 (11.4)
Drinking to intoxication days M (sd)	6.7 (8.6)	4.8 (6.5)	8.5 (10.0)

The majority of participants in the ALC-TXT-CBT condition (n = 11; 65%) opted to receive 2 text messages daily in addition to medication reminders, with the remainder having selected 3 daily messages. In terms of usage of optional features, the text CRAVE option was utilized once, and the LIFELINE and CALL options were not utilized.

### Alcohol use

**Fig 2:**
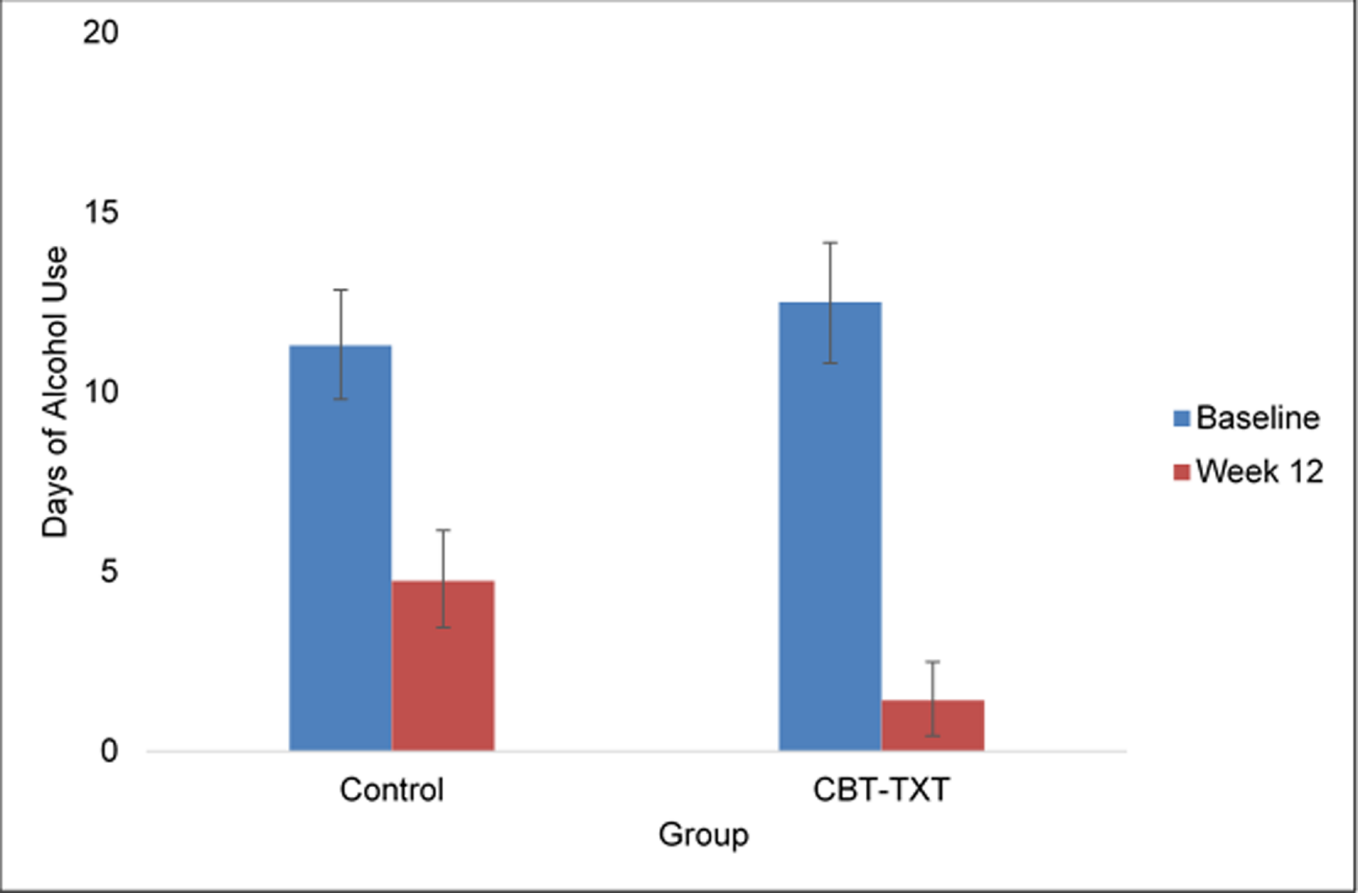
Alcohol use frequency (past 30 days) from baseline to week 12 by treatment group.

**Fig 3:**
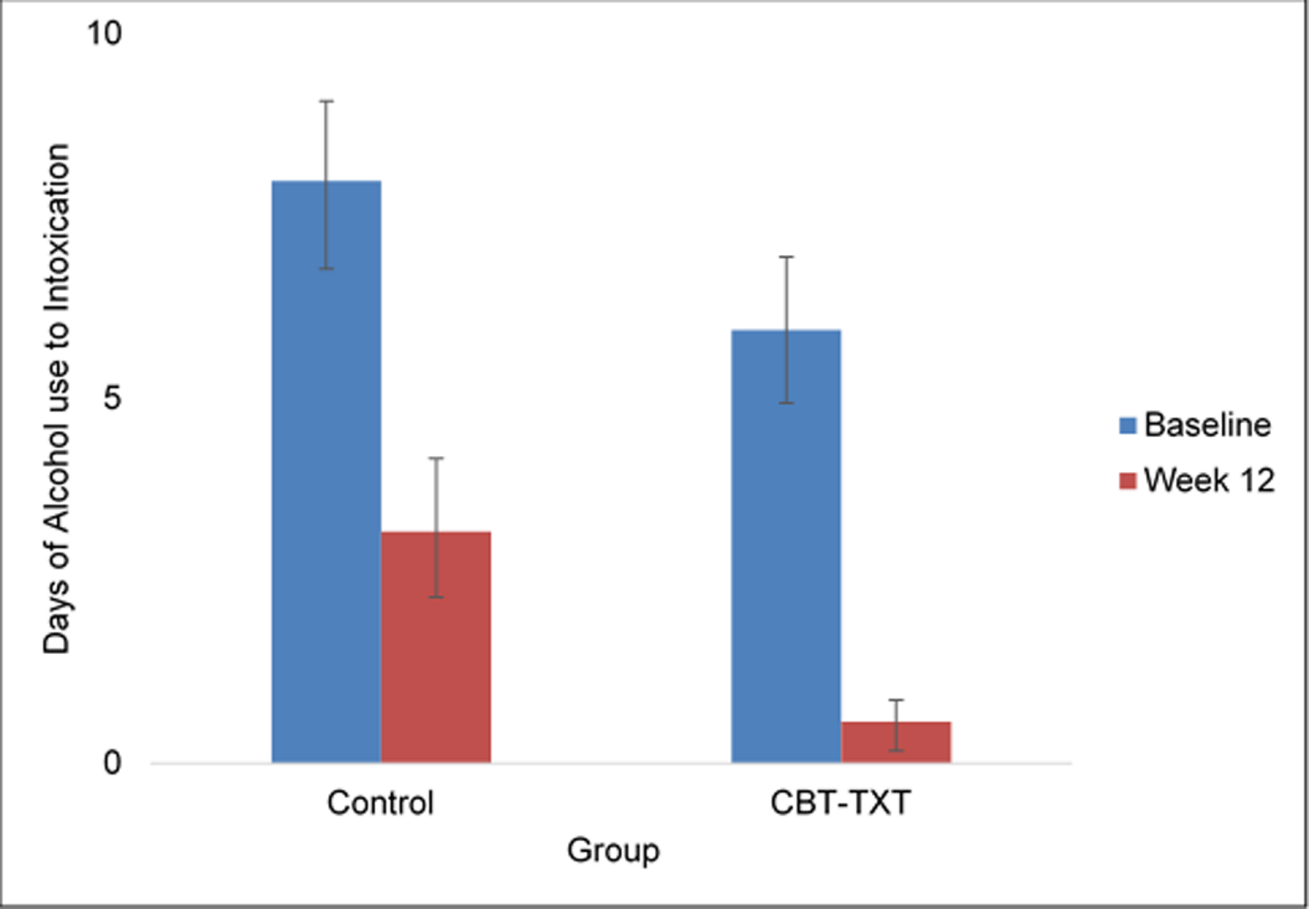
Mean days of alcohol intoxication (past 30 days) from baseline to week 12 by treatment group.

Participants in the ALC-TXT-CBT (n = 18) intervention reported fewer days of alcohol use in the past 30 at treatment-end, relative to those in the INFO condition (n = 17), (M = 1.42, SD = 2.02 versus M = 4.75, SD = 8.02), though the difference did not reach statistical significance, t(28) = 1.5, p = 0.07 (Fig 2). Nevertheless, the self-reported frequency of alcohol use to intoxication was significantly lower at the end of treatment, relative to baseline for those who received ALC-TXT-CBT (M = 0.57, SD = 1.22) compared to those in the INFO control group (M = 3.18, SD = 4.57), t(28) = 2.07, p = 0.02) (see Fig 3). There were no significant between-group differences observed in other drug use frequency (cannabis, amphetamines, cocaine and other stimulants).

### ART adherence

**Fig 4:**
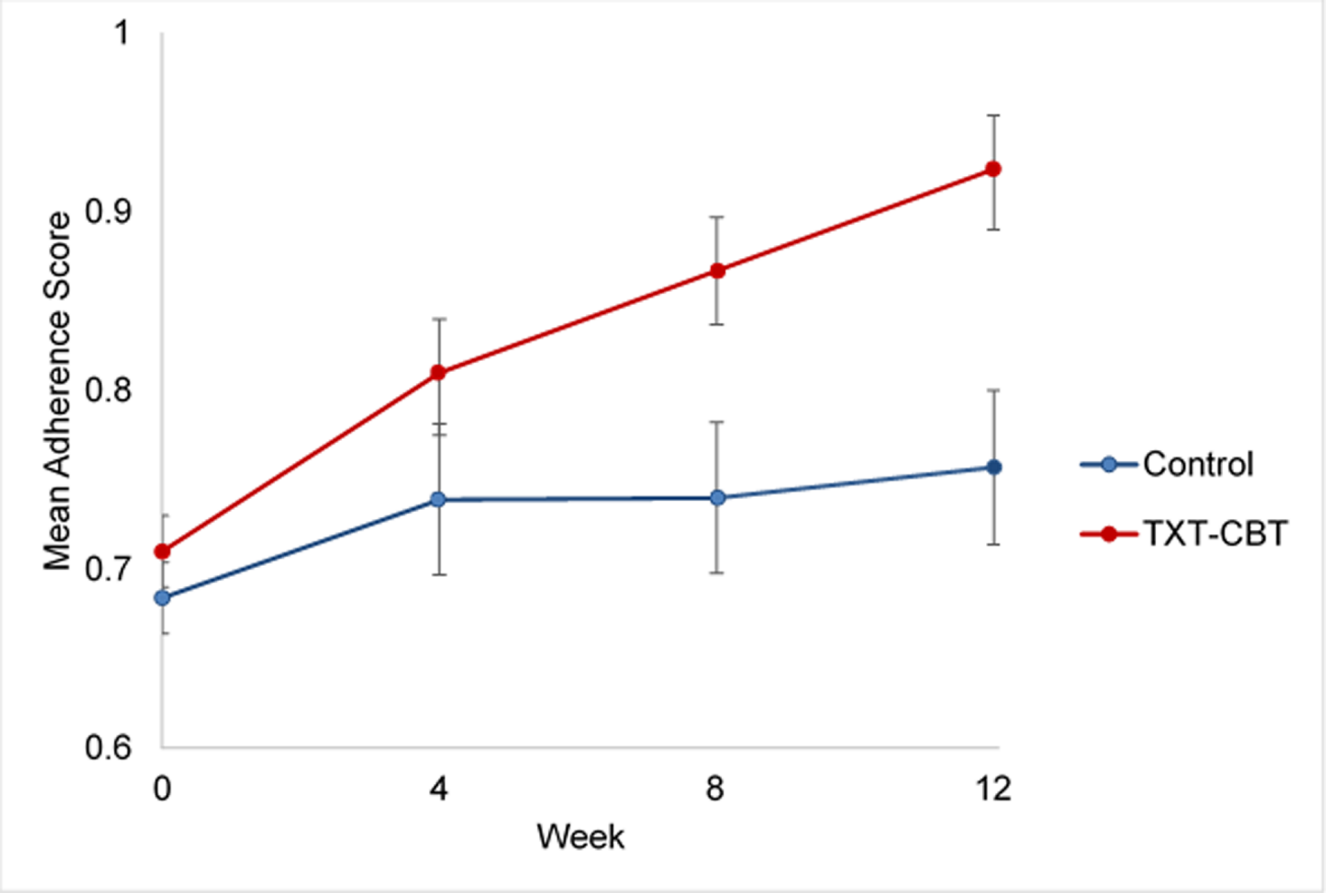
Mean ART adherence scores at weeks 0, 4, 8, and 12 by treatment group.

**Fig 5:**
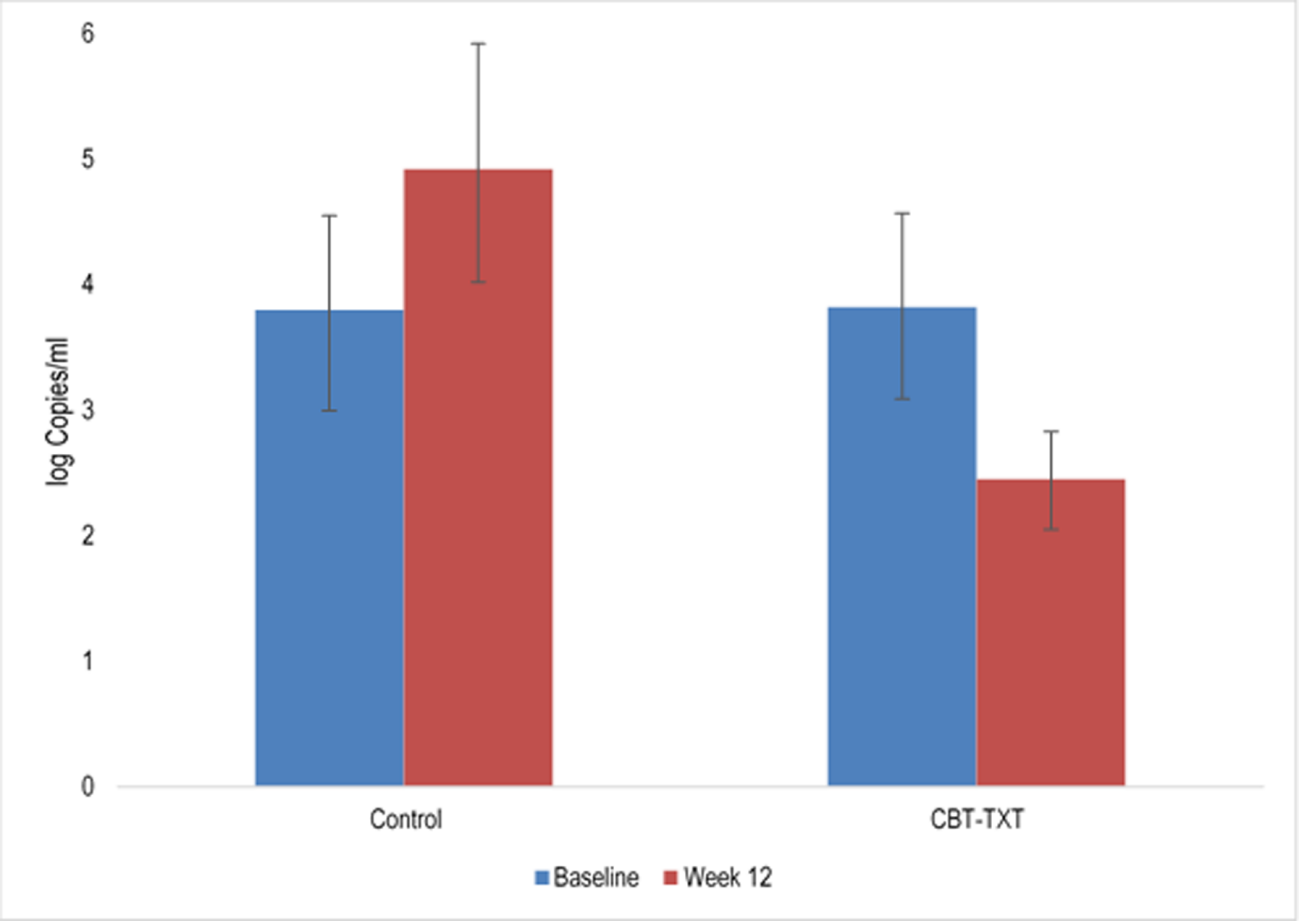
Mean log viral load levels as a function of treatment group at baseline and week 12.

Mean ART adherence scores, based upon the unannounced pill count procedures, increased significantly over the course of the 12-week intervention period among ALC-TXT-CBT participants, relative to those who received INFO (β = 0.16, p = 0.04, R^2^ = .0.19). Pairwise comparisons of adherence scores at each time point indicated that at treatment completion, ART adherence was significantly higher for those in the ALC-TXT-CBT condition (M = 0.92, SD = 0.16) relative to those in the INFO condition (M = 0.75, SD = 0.22), t(26) = 2.15, p = 0.02 (Effect Size = 0.82) (Fig 4). Consistent with adherence score data, log viral load changed differentially as a function of treatment group, as evidenced by a group x time interaction, with ALC-TXT-CBT participants demonstrating significant decreases from baseline to treatment-end (β = -2.43, p = 0.04; see Fig 5); likewise, those assigned to ALC-TXT-CBT evidenced lower log viral load (M = 1.95, SD = 2.38) than those assigned to the INFO condition (M = 4.77, SD = 4.51) at the end of treatment, t(28) = -2.04, p<0.05.

## Discussion

To our knowledge, this was the first study in which an integrated CBT intervention targeting alcohol use and ART adherence concurrently was delivered to individuals with HIV and alcohol use disorder comorbidity using text messaging. Our promising findings from the pilot RCT presented herein extend prior work supporting the use of text messaging interventions for individuals with HIV [50,51]. Despite advances in the development of text messaging approaches to promoting adherence to various aspect of HIV care, there is a paucity of behavioral mHealth interventions research on people living with HIV and comorbid alcohol and other substance use disorders [52–54]. While at least one recent study addressed this gap by recruiting vulnerable populations with one or more risk factors for disengagement from HIV care (e.g., mental illness, active substance use, poor baseline ART adherence) to receive a text messaging intervention [55], the intervention content addressed ART adherence, but not alcohol or drug use. Correspondingly, improvements were observed in ART adherence, but not substance use. Findings from the present investigation suggest that: (1) alcohol use and ART adherence can be effectively addressed using a combination of CBT-based adherence counseling and evidence-based relapse prevention techniques; (2) these techniques can be delivered using a text messaging platform requiring minimal clinician involvement; and (3) ALC-TXT-CBT not only reduces heavy alcohol use, but facilitates ART adherence in comorbid populations with alcohol use disorders and HIV.

Despite recent work demonstrating, in a large sample (N = 757), that people living with HIV who consume alcohol express a strong interest in using mobile technology to self-manage alcohol use [56], to our knowledge, this study is the first text messaging intervention trial to specifically target alcohol use among those with HIV using CBT. Although the literature describing text messaging as an intervention platform to address alcohol and illicit drug use is rapidly burgeoning [57], within the HIV treatment adherence research area, the use of text messaging in relation to alcohol use has been limited to ecological momentary assessment studies for the purposes of monitoring and understanding alcohol use patterns [12,58,59]. The current data suggest that this mode holds promise in supporting recovery from alcohol use disorders. In light of the known deleterious impact of alcohol use on ART adherence behaviors, and consequently, the clinical course and outcomes from HIV infection, effective, scalable strategies for individuals with problematic alcohol use are urgently needed. Moreover, illicit drug use is also problematic among people living with HIV [60], not only increasing rates of HIV transmission but also posing a barrier to effective disease self-management [61]. As such, innovative approaches to bolstering ART medication adherence among alcohol users living with HIV have the potential to act as extended treatment models for other types of substance users.

The CBT-based text messaging intervention literature is at an early stage of development and little is known about the characteristics of individuals who might be particularly well suited for this treatment approach. The current findings support the need for a larger intervention trial to examine factors that moderate treatment response, relative to other (i.e., clinician delivered) types of interventions. Understanding psychological process variables that are impacted by ALC-TXT-CBT will be an important means of determining the key ingredients of this approach and can inform further refinement and effective dissemination of this intervention.

### Limitations and offsetting strengths

This study had several limitations. First, alcohol use was assessed using self-report. Although this is relatively standard in the alcohol treatment literature given the brief window of time following consumption in which alcohol use can be detected via biochemical testing, self-report methods are inherently limited. Second, the INFO control condition was not matched to the texting intervention for time and attention; as such, some therapeutic change observed among ALC-TXT-CBT participants may be attributable to the increased attention conferred by this condition. In addition, because the ALC-TXT-CBT intervention incorporated both brief counseling and text messaging components, it is not possible to distinguish the effects of these elements. Third, statistical power for detecting effects of the intervention was limited due to the small sample size; as such, effects of the intervention on some target outcomes that did not reach statistical significance (e.g., overall alcohol use frequency) could not be fully determined. Replication with a larger sample in a fully powered trial is needed. Fourth, objective data concerning adherence were not measured in the research setting and thus, though extrapolated from medical records, the measurements did not correspond precisely with the study timeline., Fifth, long term, post-treatment outcomes beyond immediate termination of the intervention were not assessed and will be important as a means of understanding the durability of ALC-TXT-CBT effects on ART adherence and alcohol use. Finally, the study was conducted in an urban metropolitan city and, as such, findings may not be generalizable to substance users and people living with HIV in different geographical regions.

Several major strengths of the present study warrant comment. First, this study yielded novel insights as to how to deploy an integrated, theory- and evidence-based CBT intervention for the treatment of HIV-alcohol use disorder comorbidity via text messaging. Unlike the majority of existing text messaging intervention strategies targeting adherence behaviors, rather than implementing a single behavioral strategy (e.g., reminders or motivational sayings), ALC-TXT-CBT incorporates manualized, evidence-based content from both alcoholism and medication adherence interventions [4,62,63]. Second, ALC-TXT-CBT contains various personalized features including bidirectional communication, tailored skills training to match individually identified deficits that contribute to problematic adherence and substance use behaviors, and medication reminders matched to individuals’ ART dosing schedule. These intervention elements have been found in meta-analyses and reviews of text messaging interventions to be associated with optimal adherence outcomes in populations with HIV [51]. Third, we focused on an understudied population in the adherence intervention literature (i.e., adults living with HIV and comorbid alcohol use disorders) with an urgent need for rapidly deployable, low cost behavioral approaches to address alcohol use and suboptimal ART adherence. This model may have potential utility for other infectious diseases that are commonly comorbid with alcohol and drug use (e.g., Hepatitis C). Moreover, ART adherence outcomes were assessed via both self-report and biological measures.

## Conclusions

Findings from this pilot RCT suggest that ALC-TXT-CBT is a promising text messaging intervention strategy for adults living with HIV and comorbid alcohol use disorders. Plans for a fully powered RCT, evaluating this approach relative to a time and attention control are presently under way.

## Supporting information

S1 Data (DOCX)

S1 Checklist CONSORT 2010 checklist of information to include when reporting a randomised trial. (DOCX)

## References

[1] Egger M, May M, Chêne G, Phillips AN, Ledergerber B, Dabis F, et al Prognosis of HIV-1-infected patients starting highly active antiretroviral therapy: a collaborative analysis of prospective studies. The Lancet. 2002 7 13;360(9327):119–29. 10.1016/s0140-6736(02)09411-4 12126821

[2] Centers for Disease Control and Prevention. HIV in the United States: At a Glance. Available at https://www.cdc.gov/hiv/pdf/statistics/overview/cdc-hiv-us-ataglance.pdf. Accessibility verified April 30, 2019.

[3] Katzenstein DA. Adherence as a particular issue with protease inhibitors. Journal of the Association of Nurses in AIDS Care.1997;8(Suppl.), 10–17. 10.1016/s1055-3290(97)80003-9 9356957

[4] Safren SA, Otto MW, Worth J. Life-Steps: Applying cognitive-behavioral therapy to patient adherence to HIV medication treatment. Cognitive and Behavioral Practice. 1999;6, 332–341.

[5] Paterson DL, Swindells S, Mohr J, Brester M, Vergis EN, Squier C, et al Adherence to Protease Inhibitor Therapy and Outcomes in Patients with HIV Infection. Ann Intern Med. 2000;133:21–30. 10.7326/0003-4819-133-1-200007040-00004 10877736

[6] Bangsberg DR, Perry S, Charlebois ED, Clark RA, Roberston M, Zolopa AR, et al Non-adherence to highly active antiretroviral therapy predicts progression to AIDS. AIDS. 2000; 15(9):1181–1183. 10.1097/00002030-200106150-00015 11416722

[7] Volberding PA, Deeks SG. Antiretroviral therapy and management of HIV infection. Lancet. 2010; 376(9734):49–62. 10.1016/S0140-6736(10)60676-9 20609987

[8] Wood E, Hogg RS, Yip B, Harrigan PR, O'Shaughnessy MV, Montaner JS. Effect of medication adherence on survival of HIV-infected adults who start highly active antiretroviral therapy when the CD4+ cell count is 0.200 to 0.350 × 10(9) cells/L. Annals of internal medicine. 2003; 139(10):810–816. 10.7326/0003-4819-139-10-200311180-00008 14623618

[9] Gmel G, Shield KD, Rehm J. Developing a method to derive alcohol-attributable fractions for HIV/AIDS mortality based on alcohol's impact on adherence to antiretroviral medication. Population Health Metrics.2011; 9(1), 5 10.1186/1478-7954-9-5 21320310 PMC3048542

[10] Parsons JT, Rosof E, Mustanski B. The temporal relationship between alcohol consumption and HIV-medication adherence: A multilevel model of direct and moderating effects. Health Psychology. 2008 9;27(5):628 10.1037/a0012664 18823189 PMC2666539

[11] Kalichman SC, Grebler T, Amaral CM, McNerey M, White D, Kalichman MO, et al Intentional non-adherence to medications among HIV positive alcohol drinkers: prospective study of interactive toxicity beliefs. Journal of general internal medicine. 2013 3 1;28(3):399–405. 10.1007/s11606-012-2231-1 23065532 PMC3579979

[12] Pellowski JA, Kalichman SC, Kalichman MO, Cherry C. Alcohol-antiretroviral therapy interactive toxicity beliefs and daily medication adherence and alcohol use among people living with HIV. AIDS care. 2016 8 2;28(8):963–70. 10.1080/09540121.2016.1154134 26964014 PMC4963817

[13] Hendershot CS, Stoner SA, Pantalone DW, Simoni JM. Alcohol use and antiretroviral adherence: Review and meta-analysis. Journal of Acquired Immune Deficiency Syndromes. 2009; 52(2):180–202. 10.1097/QAI.0b013e3181b18b6e 19668086 PMC2815237

[14] Patrick K, Raab F, Adams M, Dillon L, Zabinski M, Rock CL, et al A text message-based intervention for weight loss: Randomized controlled trial. Journal of Medical Internet Research. 2009; 11(1) 1–9. PMCID: PMC2729073. 10.2196/jmir.1100 PMC2729073 19141433

[15] Franklin VL, Waller A, Pagliari C, Greene SA. A randomized controlled trial of Sweet Talk, a text-messaging system to support young people with diabetes. Diabetic Medicine. 2006; 23(12), 1332–1338. 10.1111/j.1464-5491.2006.01989.x 17116184

[16] Hurling R, Catt M, Boni MD, Fairley BW, Hurst T, Murray P, et al Using Internet and mobile phone technology to deliver an automated physical activity program: Randomized controlled trial. Journal of Medical Internet Research. 2007; 9(2), e7. 10.2196/jmir.9.2.e7 17478409 PMC1874722

[17] Puccio JA, Belzer M, Olson J, Martinez M, Salata C, Tucker D, et al The use of cell phone reminder calls for assisting HIV-infected adolescents and young adults to adhere to highly active antiretroviral therapy: A pilot study. AIDS Patient Care and STDs. 2006; 20, 438–444. 10.1089/apc.2006.20.438 16789857

[18] Song T, Qian S, Yu P. Mobile health interventions for self-control of unhealthy alcohol use: systematic review. JMIR mHealth and uHealth. 2019;7(1):e10899 10.2196/10899 30694200 PMC6371076

[19] Suffoletto B, Kristan J, Chung T, Jeong K, Fabio A, Monti P, et al An interactive text message intervention to reduce binge drinking in young adults: a randomized controlled trial with 9-month outcomes. PloS one. 2015 11 18;10(11):e0142877 10.1371/journal.pone.0142877 26580802 PMC4651466

[20] Song T, Qian S, Yu P. Mobile Health Interventions for Self-Control of Unhealthy Alcohol Use: Systematic Review. JMIR Mhealth Uhealth. 2019;7(1):e10899 Published 2019 Jan 29. 10.2196/10899 30694200 PMC6371076

[21] Quintana Y, Martorell EA, Fahy D, Safran C. A Systematic Review on Promoting Adherence to Antiretroviral Therapy in HIV-infected Patients Using Mobile Phone Technology. Applied Clinical Informatics. 2018 4;9(02):450–66. 29925099 10.1055/s-0038-1660516 PMC6010354

[22] Horvath T, Azman H, Kennedy GE, Rutherford GW. Mobile phone text messaging for promoting adherence to antiretroviral therapy in patients with HIV infection. Cochrane Database of Systematic Reviews. 2012(3). 10.1002/14651858.CD009756 PMC6486190 22419345

[23] Finitsis DJ, Pellowski JA, Johnson BT. Text message intervention designs to promote adherence to antiretroviral therapy (ART): a meta-analysis of randomized controlled trials. PLoS One. 2014;9(2):e88166 10.1371/journal.pone.0088166 24505411 PMC3914915

[24] Velthoven MV, Brusamento S, Majeed A, Car J. Scope and effectiveness of mobile phone messaging for HIV/AIDS care: a systematic review. Psychology, health & medicine. 2013 3 1;18(2):182–202. 10.1080/13548506.2012.701310 22788357

[25] Hall AK, Cole-Lewis H, Bernhardt JM. Mobile text messaging for health: a systematic review of reviews. Annual review of public health. 2015 3 18;36:393–415. 10.1146/annurev-publhealth-031914-122855 25785892 PMC4406229

[26] Morgenstern J, Kuerbis A, Muench F. Ecological Momentary Assessment and Alcohol Use Disorder Treatment. Alcohol Res. 2014;36(1):101–109. 26259004 10.35946/arcr.v36.1.10 PMC4432849

[27] Dulin PL, Gonzalez VM, Campbell K. Results of a pilot test of a self-administered smartphone-based treatment system for alcohol use disorders: usability and early outcomes. Subst Abus. 2014;35(2):168–175. 10.1080/08897077.2013.821437 24821354 PMC4019942

[28] Gustafson DH, McTavish FM, Chih MY, et al A smartphone application to support recovery from alcoholism: a randomized clinical trial. JAMA Psychiatry. 2014;71(5):566–572. 10.1001/jamapsychiatry.2013.4642 24671165 PMC4016167

[29] Riley WT, Rivera DE, Atienza AA, Nilsen W, Allison SM, Mermelstein R. Health behavior models in the age of mobile interventions: are our theories up to the task?. Transl Behav Med. 2011;1(1):53–71. 10.1007/s13142-011-0021-7 21796270 PMC3142960

[30] Safren SA, O’Cleirigh CO, Tan JY, Raminani SR, Reilly LC, Otto MW, et al A randomized controlled trial of cognitive behavioral therapy for adherence and depression (CBT-AD) in HIV-infected individuals. Health Psychology. 2009; 28, 1–10. 10.1037/a0012715 19210012 PMC2643364

[31] Safren SA, Otto MW, Worth J, Salomon E, Johnson W, Mayer K, et al Two strategies to increase adherence to HIV antiretroviral medication: Life-Steps and medication monitoring. Behavioural Research and Therapy. 2001; 39, 1151–1162. 10.1016/s0005-7967(00)00091-7 11579986

[32] Magill M, Ray LA. Cognitive-behavioral treatment with adult alcohol and illicit drug users: A meta-analysis of randomized controlled trials. Journal on Studies of Alcohol and Drugs. 2009; 70(4), 516–527. 10.15288/jsad.2009.70.516 19515291 PMC2696292

[33] Kay‐Lambkin FJ, Baker AL, Lewin TJ, Carr VJ. Computer‐based psychological treatment for comorbid depression and problematic alcohol and/or cannabis use: a randomized controlled trial of clinical efficacy. Addiction. 2009 3;104(3):378–88. 10.1111/j.1360-0443.2008.02444.x 19207345

[34] Glasner-Edwards S., Patrick K., Ybarra M. L., Reback C. J., Rawson R. A., Chokron Garneau H., et al A Cognitive Behavioral Therapy-Based Text Messaging Intervention Versus Medical Management for HIV-Infected Substance Users: Study Protocol for a Pilot Randomized Trial. JMIR research protocols. 2016; 5(2), e131 10.2196/resprot.5407 27341852 PMC4938885

[35] Glasner SG, Patrick K, Ybarra M, Reback CJ, Ang A, Kalichman S, et al Promising Outcomes from a Cognitive Behavioral Therapy Text-Messaging Intervention Targeting Drug Use, HIV Risk Behaviors, and Antiretroviral Therapy Adherence among Drug Users Living with HIV. Annals of Behavioral Medicine. 2019. Under review. 10.1016/j.drugalcdep.2021.109229 34979421

[36] Glasner, S., Venegas, A., Garneau, H.C., Ray, L., & Rawson, R.A. (2017, June). Development of a Cognitive Behavioral Therapy-based text messaging intervention for HIV+ alcohol users. Paper presented at the 40th annual meeting of the Research Society on Alcoholism (Denver, Colorado).

[37] Jairath N, Horgerney M, Parsons C. The role of the pilot study: A case illustration from cardiac nursing research. 2000 Applied Nursing Research, 13, 92–96. 10.1016/s0897-1897(00)80006-3 10842905

[38] Lancaster S, Dodd P.R., Williamson G.A. Design and analysis of pilot studies: recommendations for good practice. 2004 Journal of Evaluation in Clinical Practice, 10(2), 307–12. 10.1111/j..2002.384.doc.x 15189396

[39] Hedeker D, Gibbons R. Longitudinal Data Analysis. 2006 Hoboken, NJ: John Wiley.

[40] Schulz KF, Altman DG, Moher D. CONSORT 2010 statement: Updated guidelines for reporting parallel group randomized trials. J Pharmacol Pharmacother. 2010; 1(2), 100–7. 10.4103/0976-500X.72352 21350618 PMC3043330

[41] Miller S. M., & Diefenbach M. A. The Cognitive-Social Health Information-Processing (C-SHIP) model: A theoretical framework for research in behavioral oncology In Krantz D. S. & Baum A. (Eds.), Technology and methods in behavioral medicine (pp. 219–244). 1998 Mahwah, NJ, US: Lawrence Erlbaum Associates Publishers.

[42] Rodgers A, Corbett T, Bramley D, Riddell T, Wills M, Lin R-B, et al Do u smoke after txt? Results of a randomized trial of smoking cessation using mobile phone text messaging. Tobacco Control. 2005; 14(4), 255–261. 10.1136/tc.2005.011577 16046689 PMC1748056

[43] McLellan AT, Kushner H, Metzger D, Peters R, Smith I, Grissom G, et al The Fifth Edition of the Addiction Severity Index. J Subst Abuse Treat. 1992;9(3):199–213. 10.1016/0740-5472(92)90062-s 1334156

[44] Kalichman SC, Amaral C, Cherry C, Flanagan J, Pope H, Eaton L, et al Monitoring antiretroviral adherence by unannounced pill counts conducted by telephone: Reliability and criterion-related validity. HIV Clinical Trials. 2008; 9, 298–308. 10.1310/hct0905-298 18977718 PMC2937191

[45] Kalichman SC, Amaral C, Swetsze C, Eaton L, Kalichman MO, Cherry C, et al Monthly unannounced pill counts for monitoring HIV treatment adherence: tests for self-monitoring and reactivity effects. HIV Clinical Trials. 2010; 11(6), 325–331. 10.1310/hct1106-325 21239360 PMC7737540

[46] Giordano TP, Guzman D, Clark R, Charlebois ED, Bangsberg DR. Measuring adherence to antiretroviral therapy in a diverse population using a visual analogue scale. HIV Clinical Trials. 2004; 5(2), 74–9. 10.1310/JFXH-G3X2-EYM6-D6UG 15116282

[47] Kalichman SC, Kalichman MO, Cherry C, Swetsze C, Amaral C, White D, et al Brief behavioral self-regulation counseling for HIV treatment adherence delivered by cell phone: An initial test of concept trial. AIDS Patient Care and STDs. 2011; 25(5). 10.1089/apc.2010.0367 PMC3085946 21457056

[48] Laird NM, Ware JH. Random-effects models for longitudinal data. Biometrics. 1982 12 1;38(4):963–74. 7168798

[49] Fitzmaurice GM, Laird NM, Ware JH. Applied longitudinal analysis. John Wiley & Sons; 2012 10 23.

[50] Shoptaw S, Reback CJ, Peck JA, Yang X, Rotheram-Fuller E, Larkins S, et al Behavioral treatment approaches for methamphetamine dependence and HIV-related sexual risk behaviors among urban gay and bisexual men. Drug and Alcohol Dependence. 2015; 78,125–134. 10.1016/j.drugalcdep.2004.10.004 15845315

[51] Finitsis D. J., Pellowski J. A., & Johnson B. T. Text message intervention designs to promote adherence to antiretroviral therapy (ART): a meta-analysis of randomized controlled trials. PloS one. 2014; 9(2), e88166 10.1371/journal.pone.0088166 24505411 PMC3914915

[52] McKay J. R. The use of new communications technologies to evaluate and intervene in substance use disorders. Current behavioral neuroscience reports. 2015; 2(1), 23–29. 10.1007/s40473-014-0017-y 29527457 PMC5844699

[53] Muench F., van Stolk-Cooke K., Kuerbis A., Stadler G., Baumel A., Shao S., et al A randomized controlled pilot trial of different mobile messaging interventions for problem drinking compared to weekly drink tracking. PloS one. 2017; 12(2), e0167900 10.1371/journal.pone.0167900 28146560 PMC5287456

[54] Reback C. J., Fletcher J. B., Swendeman D. A., & Metzner M. Theory-Based Text-Messaging to Reduce Methamphetamine Use and HIV Sexual Risk Behaviors Among Men Who Have Sex with Men: Automated Unidirectional Delivery Outperforms Bidirectional Peer Interactive Delivery. AIDS and behavior. 2019; 23(1), 37–47. 10.1007/s10461-018-2225-z 30006792 PMC6330245

[55] King E, Kinvig K, Steif J, Qiu AQ, Maan EJ, Albert AY, et al Mobile text messaging to improve medication adherence and viral load in a vulnerable Canadian population living with human immunodeficiency virus: a repeated measures study. Journal of medical Internet research. 2017;19(6):e190 10.2196/jmir.6631 28572079 PMC5472843

[56] Sharpe JD, Zhou Z, Escobar-Viera CG, Morano JP, Lucero RJ, Ibañez GE, et al Interest in using mobile technology to help self-manage alcohol use among persons living with the human immunodeficiency virus: A Florida Cohort cross-sectional study. Subst Abus. 2018 1 2;39(1):77–82. 10.1080/08897077.2017.1356793 28723300 PMC5775061

[57] Tofighi B, Nicholson JM, McNeely J, Muench F, Lee JD. Mobile phone messaging for illicit drug and alcohol dependence: A systematic review of the literature. Drug Alcohol Rev. 2017 7;36(4):477–491. 10.1111/dar.12535 28474374

[58] Bonar EE, Cunningham RM, Collins RL, Cranford JA, Chermack ST, Zimmerman MA, et al Feasibility and Acceptability of Text Messaging to Assess Daily Substance Use and Sexual Behaviors among Urban Emerging Adults. Addict Res Theory. 2018;26(2):103–113. 10.1080/16066359.2017.1310205 29632458 PMC5889069

[59] Pellowski JA, Kalichman SC, Cherry S, Conway-Washington C, Cherry C, Grebler T, et al The Daily Relationship Between Aspects of Food Insecurity and Medication Adherence Among People Living with HIV with Recent Experiences of Hunger. Ann Behav Med. 2016 12;50(6):844–853. 10.1007/s12160-016-9812-x 27333898 PMC5127764

[60] Des Jarlais D. C., Kerr T., Carrieri P., Feelemyer J., & Arasteh K. HIV infection among persons who inject drugs: ending old epidemics and addressing new outbreaks. AIDS. 2016; 30(6), 815–26. 10.1097/QAD.0000000000001039 26836787 PMC4785082

[61] Lucas GM. Substance abuse, adherence with antiretroviral therapy, and clinical outcomes among HIV-infected individuals. Life sciences. 2011; 88(21–22):948–952. 10.1016/j.lfs.2010.09.025 20888839 PMC3027844

[62] Rawson RA, Shoptaw SJ, Obert JL, McCann MJ, Hasson AL, Marinelli-Casey PJ, et al An intensive outpatient approach for cocaine abuse treatment. The Matrix model. J Subst Abuse Treat. 1995;12(2):117–127. 10.1016/0740-5472(94)00080-b 7623389

[63] Ling W., Hillhouse M., Ang A., Jenkins J., & Fahey J. (2013). Comparison of behavioral treatment conditions in buprenorphine maintenance. Addiction (Abingdon, England). 2013; 108(10), 1788–1798. 10.1111/add.12266 PMC3866908 23734858

