# Supplementary material for: Preliminary Efficacy of a Cognitive Behavioral Therapy Text Messaging Intervention Targeting Alcohol Use and Antiretroviral Therapy Adherence: A Randomized Clinical Trial
Source: PLoS One. 2020 Mar 12;15(3):e0229557. doi: 10.1371/journal.pone.0229557 (PMC7067560; doi:10.1371/journal.pone.0229557)
Supplement: S1 Data — (DOCX) [file pone.0229557.s001.docx]

| **UCLA Integrated Substance Abuse Programs** |
| --- |
| **Cell Phone Technology Targeting ART Adherence and Alcohol Use** |
| **Standard Operations Manual** |

Principal Investigator:

**Suzette Glasner, Ph.D**

Funding Agency:

**National Institute on Alcohol Abuse and Alcoholism (NIAAA)**

**National Institutes of Health**

## Table of Contents

1. [Study Synopsis 4](#_bookmark0)
   1. [Study Objectives 4](#_bookmark1)
   2. [Study Design 4](#_bookmark2)
   3. [Study Population 4](#_bookmark3)
   4. [Study Treatment 4](#_bookmark4)
   5. [Study Schema 6](#_bookmark5)
   6. [Schedule of Assessment Table 7](#_bookmark6)
   7. [Commonly Used Abbreviations 7](#_bookmark7)
   8. [Pre-Intervention Procedures 8](#_bookmark8)
   9. [Staffing 8](#_bookmark9)
   10. [Training 8](#_bookmark10)
   11. [Standard Operation Procedures 8](#_bookmark11)
   12. [Assembling and Maintaining Participant Files 8](#_bookmark12)
       1. [Regulatory Binder Table of Content 9](#_bookmark13)
   13. [Assembling and Maintaining Participant Files 10](#_bookmark14)
       1. [Participant Name File 10](#_bookmark15)
       2. [Participant Data File 10](#_bookmark16)
       3. [Progress Notes 11](#_bookmark17)
       4. [Screen Failures File 11](#_bookmark18)
   14. [Supplies 11](#_bookmark19)
       1. [Lab Supplies 11](#_bookmark20)
       2. [Research Supplies 11](#_bookmark21)
   15. [Participant Compensation 12](#_bookmark22)
       1. [Gift Card Storage Information 13](#_bookmark23)
   16. [IRB Approval 13](#_bookmark24)
   17. [Documentation and Record Keeping 13](#_bookmark25)
   18. [Documentation Basics 13](#_bookmark26)
   19. [Error Correction 13](#_bookmark27)
   20. [Protocol 14](#_bookmark28)
   21. [Case Report Forms (CRFs) 14](#_bookmark29)
       1. [Completing CRFs and Source Documents 14](#_bookmark30)
       2. [Scanning Completed CRFs 15](#_bookmark31)
   22. [Source Documents 15](#_bookmark32)
   23. [Progress Notes 15](#_bookmark33)
   24. [Study Logs 16](#_bookmark34)
       1. [Master Enrollment Log 16](#_bookmark35)
   25. [Document Storage 17](#_bookmark36)

[4.0 Study Interventions 17](#_bookmark37)

- 1. [Study Research Procedures 17](#_bookmark38)
  2. [Screening and Baseline Assessments (Week 0) 17](#_bookmark39)
     1. [Screening Visit 18](#_bookmark40)
     2. [Confirming Study Eligibility 19](#_bookmark41)
     3. [Handling Failed Screeners 20](#_bookmark42)
     4. [Scheduling 21](#_bookmark43)
     5. [Baseline Assessments 21](#_bookmark44)
  3. [Random Assignment 21](#_bookmark45)
  4. [Phase 3: Intervention (Weeks 4, 8 and 12) 22](#_bookmark46)
  5. [Missed Visits 23](#_bookmark48)
  6. [Biological Assessments 23](#_bookmark49)
  7. [Urine Drug Screens 23](#_bookmark50)
  8. [Urine Drug Screens 26](#_bookmark51)
  9. [Participant Discontinuation/Termination 26](#_bookmark52)
  10. [Criteria for Termination 26](#_bookmark53)

[7.1.1 Participant Incarceration 26](#_bookmark54)

- 1. [Procedures for Discontinuation 27](#_bookmark55)
  2. [Recruitment and Follow-up Topics 27](#_bookmark56)
  3. [Recruitment 27](#_bookmark57)
  4. [Retention Techniques 27](#_bookmark58)
  5. [Lost to Follow-up 28](#_bookmark59)
  6. [Data, Safety, and Monitoring 28](#_bookmark60)
  7. [Adverse Events (AEs) 28](#_bookmark61)
  8. [Serious Adverse Event (SAEs) 29](#_bookmark62)
  9. [Confidentiality 29](#_bookmark63)
     1. [Certificate of Confidentiality 29](#_bookmark64)
     2. [Breach of Confidentiality 29](#_bookmark65)
     3. [Maintaining Confidentiality 29](#_bookmark66)
     4. [Exceptions to Confidentiality 30](#_bookmark67)
  10. [Release of Information 31](#_bookmark68)
  11. [Emergencies 31](#_bookmark69)
      1. [Suicidal Participants 31](#_bookmark70)
      2. [Homicidal Participants 33](#_bookmark71)
      3. [Reporting Child/Elder/Dependent Adult Abuse 33](#_bookmark72)
  12. [Regulatory Affairs 35](#_bookmark73)
  13. [Protocol Violations 35](#_bookmark74)

## Study Synopsis

The proposed study, for alcohol dependent adults with HIV-infection, is a pilot randomized controlled trial (RCT) examining the outcomes of a 12-week behavioral support program delivered via text-messaging. It is expected that the text messaging intervention will reduce alcohol use and improve adherence to medication.

To test the effects of the intervention on these target outcomes, 25 participants receiving the text messaging intervention will be compared to 25 participants receiving an informational pamphlet. The pamphlet will contain information about the importance of HIV treatment adherence, reducing HIV risk behaviors, and health consequences associated with alcohol use.

By providing support to maximize HIV treatment regimen adherence, coupled with coping skills to promote abstinence from alcohol, the text messaging intervention may provide a promising, cost- effective, and easily deployable behavioral support program for alcohol users who are HIV-infected.

## 1.1 Study Objectives

**The aims of this study are to:**

- 1. **Implement and evaluate a 12-week cognitive behavioral therapy (CBT) intervention using text messaging via mobile phone technology (TXT-CBT) to reduce alcohol use, reduce HIV-risk behaviors and facilitate medication adherence in a population of alcohol dependent adults with HIV-infection**
  2. **Examine potential mechanisms of action of TXT-CBT. We hypothesize that TXT-CBT will produce greater reductions in alcohol use and HIV-risk behaviors, and will improve HIV treatment regimen adherence, relative to the** **control condition (pamphlet).**

## Study Design

The proposed pilot study will include 50 individuals seeking treatment for alcohol dependence, assessed according to DSM-IV criteria, and help to improve adherence to medications while reducing risk-behaviors. After satisfying all inclusion/exclusion requirements, participants will be enrolled into the study for a 12 week intervention. Participants will either be randomized to the TXT-CBT condition (n=25) or to the informational pamphlet condition (n=25).

## Study Population

## Study Treatment

*Pre-screen and informed consent.* Interested individuals will contact study personnel by phone or email. A research assistant will give interested individuals a study description over the phone. Should the individual still be interested after the study description, he/she will be pre-screened over the phone by a research assistant prior to scheduling an initial interview to begin the informed consent process. Those who pre-screen successfully will be scheduled to come to the clinic to provide informed consent and undergo additional screening to determine eligibility. Individuals who are not eligible for the study based on this initial prescreen can be referred to a different study, if appropriate.

*Diagnostic Criteria***.** In the proposed study, only those with Alcohol Dependence and HIV infection will be included. DSM-IV Alcohol Dependence diagnoses will be based on the DSM-IV Checklist diagnostic interview. The MINI Diagnostic Interview will be used to assess the presence of clinically significant psychiatric symptoms as such as psychosis or acute mania that would require ongoing treatment or make study compliance difficult. Participants will not be excluded for Axis II disorders

(e.g., Antisocial Personality Disorder) nor for co-existing Axis I disorders. Individuals unaware of their HIV status will be given a referral sheet of testing sites should they decide to get tested.

Participants unaware of their HIV status will need to get tested and should be able to provide the study staff with proof of their HIV+ diagnosis to be eligible for the study. Study staff can provide interested participants with a list of HIV testing resources/locations.

*Shortcode capability.* After providing informed consent and meeting the DSM-IV criteria for alcohol dependence, potential participants will be asked to text the word “test” to 843438 to determine whether their phone supports shortcode messages. If they receive the following message from Santech “test text received successfully” then their phone supports shortcode messages and the research assistant can proceed with the baseline assessments. However, eligibility for the study is still pending until the baseline adherence score is obtained. If their phone does not support shortcode then participants are asked to contact their service provider to enable shortcode messaging on their phone. If the issue is resolved then the potential participant can proceed with the baseline assessments and pillcounts. If the service provider cannot enable shortcode messaging then the individual cannot qualify for the study and no pillcounts are necessary.

*Baseline adherence.* To meet eligibility criteria, potential participants also need to have an adherence score < 90%. Two unannounced pillcounts done at a two week interval will be performed over the phone to collect the data needed to calculate the adherence score (see pillcount protocol). If a potential participant has a baseline adherence score that is ≥ 90% then a third unannounced pillcount is performed two weeks after the second pillcount and a new adherence score is calculated. If this score is < 90% then the individual is eligible for the study given that the other criteria for inclusion are met. If this second adherence score is ≥ 90% then the individual is not eligible to participate in the study, even if the other inclusion criteria are met.

*Randomization.* If the three inclusion criteria are met (alcohol dependence, cell phone that supports shortcode messaging and adherence < 90%) then the individual can be randomized using the randomization application on the DMC website [(http://ww](http://www.uclaisap.org/dmc/))w[.uclaisap.org/dmc/).](http://www.uclaisap.org/dmc/)) Upon randomization, participants are given the gift card breakdown letter that corresponds to their assignment.

## TXT-CBT

Participants randomized to TXT-CBT will meet for 60-90 minutes with a CBT-trained clinician to review the 12 Life-Steps concepts. In this session, the 3 most applicable medication adherence skills will be identified for emphasis in tailored messages. Participants will provide their cell phone information to the RA for programming of the intervention. These participants will also be given a treatment manual and a wallet card containing both technical instructions and descriptions of core therapeutic content/topics for each week.

A research assistant will meet with the participants monthly at data collection visits throughout the intervention phase to answer any technical questions and ensure that the intervention program is working properly. Participants will also be called monthly for unannounced pill count to evaluate adherence and weekly to collect qualitative data about their text messaging experience.

## Informational Pamphlet

Those assigned to the Informational pamphlet condition (control) will receive a pamphlet about alcohol and drug use and HIV risk behaviors. Participants in this condition will meet monthly with an RA for data collection. Participants will also be called monthly for unannounced pill count to evaluate adherence.

**Baseline Assessment:**

## Study Schema


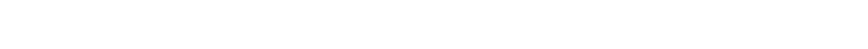


**50 alcohol dependent adults with HIV infection**


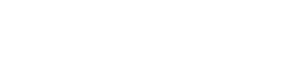


**TXT-CBT (n=25)**


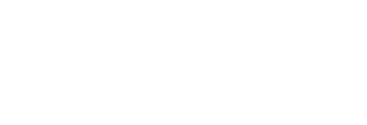


**Random Assignment** Following DSM-IV, MINI, shortcode and adherence evaluation


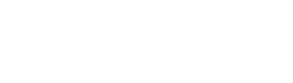


**Control (Pamphlet) (n=25)**

| Depression/Anxiety | Demographics | Viral Load/CD4 | PACS |
| --- | --- | --- | --- |
| Urine drug screen | TLFB 90 days | Self-Efficacy | Vivitrol Qs |
| HIV-Risk Behaviors | Health Status | Breathalyzer |  |
| HIV Medication Adherence | Drug Taking Confidence | Baseline SEAS |  |

**During-Treatment Assessment (every 4 weeks):** Depression/Anxiety symptoms HIV Medication Adherence PACS Drug Use, past 30 days Viral load/CD4 SEAS

Urine drug screen Breathalyzer TLFB

**End of Treatment:**

Depression/Anxiety

HIV-Risk Behaviors

HIV Medication Adherence Drug Taking Confidence

Self-Efficacy

Substance Use, past 90 days

Health Status Viral Load/CD4 Urine drug screen

Breathalyzer

PACS

SEAS

## Schedule of Assessment Table

| **Assessments** | **Baselines** | **Week 4** | **Week 8** | **Week 12** |
| --- | --- | --- | --- | --- |
| MINI | X |  |  |  |
| DSM-IV Checklist | X |  |  |  |
| DSM-IV Checklist Craving question | X |  |  |  |
| PHQ9 | X | X | X | X |
| OASIS | X | X | X | X |
| Risk Behavior Survey | X |  |  | X |
| Breathalyzer | X | X | X | X |
| Urine drug screen | X | X | X | X |
| Penn Alcohol Craving Scale | X | X | X | X |
| Subjective Effects of Alcohol Scale | X | X | X | X |
| ASI | X |  |  | X |
| TLFB | X | X | X | X |
| Medical Marijuana | X |  |  |  |
| Socrates A | X |  |  | X |
| SF-12 | X |  |  | X |
| Drug-Taking Confidence | X |  |  | X |
| Self-Efficacy | X | X | X | X |
| Vivitrol Questions | X | X | X | X |
| ART unannounced pill count | X | X | X | X |
| Viral Load/CD4 | X |  |  | X |
| Medication Compliance (self-efficacy) | X | X | X | X |
| Behavioral Strategies | X |  |  | X |
| Ancillary Treatments | X | X | X | X |
| Concomitant Medications | X | X | X | X |
| Weekly qualitative calls |  | X | X | X |
| Tailoring variables | X |  |  |  |

## Commonly Used Abbreviations

TXT- Text or texting

CBT-Cognitive behavioral therapy ART-Antiretroviral therapy

RA- Research assistant

## Pre-Intervention Procedures

## Staffing

## Training

All study staff will be appropriately trained in the following areas prior to participating in any research-related activities:

- - - Good Clinical Practice (GCP)
    - Human Subjects/HIPAA (UCLA IRB)
    - Protocol-specific training for research staff
    - Protocol-specific training for study medical staff
    - Data Collection/CRF Completion

## Standard Operation Procedures What is an SOP?

A Standard Operating Procedure, or SOP, gives very detailed and specific instructions for carrying out a procedure at your site. For example, this Standard Operations Manual is intended to instruct research staff on how to conduct this study.

## Why do we need SOPs?

SOPs are needed to make sure that research procedures are carried out in an exact, pre- determined way. They help to make sure that research is conducted consistently. Ideally, if you had to be away from work for several days, someone else could perform your duties by reading the protocol, the SOM, and SOPs.

## Assembling and Maintaining Participant Files

The Regulatory Files, also known as the Regulatory Binder or Essential Study Documents Files, are designed to facilitate regulatory compliance, effective monitoring and study document maintenance.

The Regulatory Files contain, or indicate the location of, documents that allow for:

- The reconstruction of the study and trial management,
- The evaluation of the study and data quality and validity, and
- Verification of regulatory compliance through monitoring and auditing of the study.

The Regulatory Files include documents such as the protocol, investigator’s brochure, case report forms, source documents, regulatory and Institutional Review Board documents, informed consents, and study correspondence.

This document provides direction and organization for the development and maintenance of the regulatory files (Essential Study documents) for this study.

The Regulatory Files format contains 12 main sections. Each main section of the Regulatory Files is divided from the other sections by a binder tab and includes a list of documents and their descriptions. Subsections may also be separated by tab dividers or by colored card stock or paper, or they may have no formal separation from other documents within a section.

To ensure consistency and standardization, contents are organized in reverse chronological order. As the contents exceed the capacity of one binder or file box, additional binders/boxes will be added. The order of the main sections can be altered to fit the binders or file boxes as needed. The Master Regulatory Files will be located at the study site.

## 2.4.1 Regulatory Binder Table of Content

1. Contact Information

Study Personnel Contacts SAE/AE Contacts

1. Protocol

Current Protocol Protocol Signature Page Change Logs

Obsolete Protocol(s)

Standard Operations Manual (SOM) SOPs (Protocol & Local Facility) Protocol Violation Procedures Protocol Violation Reports/Logs Other:

1. Study Intervention

Investigator Brochure Medication Management Medication Shipping Invoices Dispensing Records Disposition Records Randomization Procedures

Psychosocial Treatment Manuals Audio/Video Recordings

Other:

1. Biological Measures (BM) BM Instructions BM Normal Values BM Records

BM/Lab Certifications BM Shipping Invoices BM/Lab Communication Other:

1. Participant Information Research Records CRFs as Source

Case Report Forms (CRFs) CRF Instruction Manual Study Logs

Participant Compensation Other:

1. Research Personnel

Staff Signature Log Resumes/CVs

Licensure/Certifications DEA Certificates Training Documents

1. Consents

Current Consent(s)

Other Participant Agreements Obsolete Consents

Signed Consents

1. Regulatory

Form 1572

Certificate of Confidentiality (CoC) Limits of Confidentiality

Other Study Approval Documents Other:

1. Institutional Review Board (IRB) IRB Protocol Approvals Other IRB Approvals Advertising/Recruitment IRB Correspondence

IRB Roster/FWA

1. Safety Reporting

AE/SAE Procedures SAE Reports

DSMB Reports Other:

1. Quality Assurance (QA) Site Visit Log

QA Monitor Reports Other Monitoring Reports QA Correspondence

1. Correspondence

Study Related Correspondence Meeting Minutes

Other:

The Regulatory Files are considered part of the study record. All study documents will be kept for a minimum of 7 years before destroying. If the study records are stored at an off site location, an inventory of the documents stored off site and their location must be maintained.

## Assembling and Maintaining Participant Files

Participant files should be stored in files or binders. It must be determined who is primarily responsible for maintaining the files, though in most cases it makes the most sense for the Research Assistants (RAs) to perform this task. There are a few guidelines for this study:

- - - All participant files are legal documents and the information they contain is strictly confidential. At no time should participant files, including source documents/CRFs or other study materials, leave the study clinic. In other words, files should not be taken home or left in any location other than a secure environment.
    - Certain rare circumstance may involve removing a file from the study clinic. In such cases, files MUST be transported in a locked, secured bag or container.
    - Participant information MUST be stored within a locked file cabinet (or similar arrangement) within a designated locked office. Typically, only research staff has access to the file cabinets.

**A Note About Storing Participant Names and IDs Separately**: Federal regulations dictate only that patient confidentiality is maintained, but not that names and participant IDs be kept separate. That said, it is most protective of confidentiality if names and IDs are kept separate, and files will be organized accordingly wherever possible. For example, files linking names with participant IDs such as the Master Enrollment Log will not be stored in the same file cabinet as the research data files (progress notes, source documents). Prior to the first enrollment, week-by-week packets will be created that contain all of the forms needed for the study. Study staff will complete the corresponding weekly packet of blank forms and then add it to the participant file. This will permit easy access to copies for creating new participant files, and it will ensure the use of the most recent version of each form. To assure confidentiality, at least two separate files for each participant, the Name File and the Research Data File(s), will exist.

## Participant Name File

This file is a single file for each participant. The Name File contains identifying information and is stored separately from the Research Data File. *Participant ID #s should not be located anywhere in the Name File*. Examples of what goes in the Name File are:

- - - Signed Informed Consent form
    - Locator form (should be updated at each visit and at each contact with participant)
    - Reimbursement-related documents, like voucher stubs, receipts, etc.
    - Any Release of Information forms
    - Any other documents containing names

## Participant Data File

These files contain all source documents and study progress notes for each participant. *The participant’s name should not be located anywhere in this file*. It is helpful to have this file organized by visit.

# Participant Data File Table of Contents

- - - - 1. Progress Notes
        2. Screening/Baseline Assessments
        3. Week 4 Assessments
        4. Week 8 Assessments
        5. Week 12 Assessments
        6. Follow-up Assessments

## Progress Notes

All study visits will have accompanying progress notes documenting the study staff members’ contact with the participant. No identifying information, such as names, should be contained in the progress notes. Progress notes can be filled out in patients’ records on the DMC website under the tab “progress notes.” Progress notes are very important to protocol documentation. Progress notes should summarize what happened, what didn’t happen that should have, and plans for the next study visit. Progress notes for this study contain a checklist of required procedures that should occur during the visit, and serve as a reminder and confirmation that study procedures were completed per protocol. Progress notes should be concise, professional, and informative, should be signed/dated, and should NOT contain the participant’s name. Information in progress notes is both clinical and research in nature.

The following are the mandatory progress notes for this study:

- Consent progress note
- Screening/Baseline visit checklist & progress note
- Randomization visit checklist & progress note
- Monthly visit checklist & progress notes
- Monthly unannounced pill count checklist & progress note
- Follow-up checklist & progress notes
- Generic progress note (to be used as needed)
- Missed Visit progress note (to be used as needed)

Always write progress notes immediately after the visit or as soon as possible thereafter. *They should always be completed for each study visit, even if the visit was missed.* If a visit must be divided into multiple days (e.g., the assessments during Screening/Baseline are done over the course of 2 days), the date each procedure was completed needs to be documented on the note. You can type your progress note using electronic copies of the templates, but do NOT save to your computer as this poses a confidentiality risk. Simply print out your note, and ALWAYS remember to sign, date, and file in the participant’s file. Progress note documentation is discussed in Section 3.6 of this manual.

## Screen Failures File

A record will be kept of all participants who are consented but later found ineligible for participation. These individuals’ data will be kept in one “Screen Failures” file.

## Supplies

## Lab Supplies

From Cliawave

- Specimen cups and test strips for urine drug screen
- Breathalyzer

## Research Supplies

Research staff will be responsible for maintaining the following supplies:

- Currently approved protocol
- Currently approved Consent Form
- Current Standard Operations Manual (SOM) including all appendices (e.g., logs, progress note templates, visit window calculator, etc.)
- Binders or folders for Regulatory documents (typically two to three 3” binders will be needed)
  - Binders or folders for Participant Name Files
  - Extra binders or folders to store various study logs
  - Research computers
  - Telephones
  - Calendar (or equivalent system) to track participant visits and procedures due
  - Miscellaneous office supplies
  - Locking file cabinets (or equivalent) for participant file storage

## 2.7 Participant Compensation

For the 25 participants assigned to the **TXT-CBT** condition the compensation (if 100% compliant) will be as follow with a maximum total of $420 to be distributed in gift cards.

Baselines:

| For data collection | $40 |
| --- | --- |
| For 1st pillcount | $20 |
| For 2nd pillcount (two weeks after first pillcount) | $20 |
| For 3rd pillcount (if necessary) (two weeks after second pillcount) | $20 |
| For providing us your latest lab work | $20 |
| To help offset the cost of texting | $20 |

Weeks 4, 8 and 12

| For data collection (per data collection) | $20 |
| --- | --- |
| For pillcount (per pillcount) | $20 |
| To help offset the cost of texting (monthly)  For providing us your latest lab work (week 12 only) | $20  $20 |

Weeks 16 & 20 pillcounts $20 (each)

Week 24, Follow-up

| For data collection | $40 |
| --- | --- |
| For week 24 pillcount | $20 |
| For providing us your latest lab work | $20 |

For the 25 participants assigned to the **CONTROL** condition (if 100% compliant) will be as follow with a maximum total of $360 in gift cards.

Baseline

| For data collection | $40 |
| --- | --- |
| For 1st pillcount | $20 |
| For 2nd pillcount (two weeks after first pillcount) | $20 |
| For 3rd pillcount (if necessary) (two weeks after second pillcount) | $20 |
| For providing us your latest lab work | $20 |

Weeks 4, 8 and 12

| For data collection | $20 |
| --- | --- |
| For pillcount | $20 |
| For providing us your latest lab work (week 12 only) | $20 |

Weeks 16 & 20 pillcounts $20 (each)

Week 24, Follow-up

| For data collection | $40 |
| --- | --- |
| For week 24 pillcount | $20 |
| For providing us your latest lab work | $20 |

Participants can also be given up to 5 bus tokens or a 5$ gas card, at the discretion of the research assistant, to offset transportation cost.

## 2.7.1 Gift Card Storage Information

Per UCLA policy, gift cards used for research purposes (in our case for participant compensation and contingency management incentives) are considered same as cash and must be stored in a safe. In addition, a log book must be maintained documenting all gift cards received and disbursed. The Gift Card Log template can be found in the appendix.

The bulk supply of gift cards for this study will be kept in a safe located in the project coordinator’s office. The safe utilizes a double-lock system (key/combination). The key lock will remain unlocked and will not be used for the purposes of this study. However, in the event that the key lock becomes locked, the key is located in Sandy’s office with the other spare keys. The combination lock will be the primary security mechanism used for our purposes. The Gift Card Logs will be kept in a 3-ring binder in the project coordinator’s office file cabinet (locked).

## 2.8 IRB Approval

The Institutional Review Board (IRB) must approve the current version of the protocol, consent form, advertising materials, and assessment instruments. Throughout the duration of the study, new versions of the protocol may be created or assessment instruments may be added or altered. In both cases, an amendment to the IRB must be submitted in order to obtain approval for these updates before implementing them. In addition, changes to the consent form require IRB approval.

## Documentation and Record Keeping

## Documentation Basics

Concerns about the safety and welfare of participants in clinical trials and the integrity of study data necessitated the concept of Good Clinical Practice (GCP). A critical component of GCP is study documentation. Documentation includes all records, in any form, that describe or record the methods, conduct, and/or results of a trial, the factors affecting a trial, and actions taken. Appropriate documentation ensures that any questions that arise regarding clients’ participation can be answered. Timely and accurate documentation also ensures that the data are complete and as free from errors as possible.

Documentation should be complete, accurate, and thorough. Study documentation includes all data collection forms (electronic and/or paper), workbooks, CRFs, source documents, monitoring logs and appointment schedules, correspondence and regulatory documents (e.g., signed protocol and amendments, IRB correspondence and approved consent form and signed informed consent forms, Statement of Investigator form, and clinical supplies receipt and distribution records).

## Error Correction

Everyone makes errors. The important thing is to know what to do about errors so that they do not create questions about the integrity of the data.

**MOST IMPORTANTLY** – If you make a mistake and don’t know what to do, consult with your supervisor. Trying to guess your way through a mistake will probably make it look like you are trying to cover something up. Almost all mistakes can be handled, but it is important to deal with them directly.

## If you make a mistake while writing something down, put a single line through it, initial and date beside that line, and then put in the correct information. For example:

3/27/06 Participant was seen by study physician BT 03/27/06 nurse practitioner to have liver test blood sample collected. Bill Thomason

*Never* cover up your mistakes by:

- - - Scribbling over the mistake
    - Using White-Out or similar product
    - Re-writing the note

All of the study data will be entered and maintained in one database. Data checks will be run on study data throughout the trial, and research staff may receive queries for data that is inconsistent or needs clarification. Research staff is expected to resolve data queries within 14 days.

## Protocol

According to Good Clinical Practice (GCP) standards, a protocol is “a document that describes the objective(s), design, methodology, statistical considerations, and organization of a trial.” A current copy of the protocol approved by the Institutional Review Board is an essential component of the study documents and should be included in the Regulatory Binder.

If changes are made to the protocol during the course of the trial, a protocol amendment must be developed and submitted to the Institutional Review Board for approval. A protocol amendment “is a written description of a change(s) to or formal clarification of a protocol.” In the event that a protocol amendment is made and approved by the Institutional Review Board, the now obsolete protocol should be filed in the Obsolete Protocols section of the Regulatory Binder to ensure that the study staff is referring to the approved, current copy of the protocol to answer any questions.

## Case Report Forms (CRFs)

Case Report Forms (CRFs) are the complete record of all data collected from the research participants during their participation in the protocol. The CRF is a electronic or printed document that includes surveys, questionnaires, interviews, laboratory reports, etc. CRFs are an efficient way of organizing information and ensuring that all of the information that is to be collected from a participant is actually collected according to the schedule specified in the protocol. Additionally, the CRF provides an organizational system for storing the data. The CRFs are contained in the appendix.

## Completing CRFs and Source Documents

When completing CRFs, you should:

- - - Use black ink only
    - Write clearly
    - Correct mistakes by crossing out the incorrect response with a single line then initialing and dating the correction
    - Never cover up mistakes by writing over them or using white out

When collecting all data, you should:

- - - Ask every question
    - Ask the questions as they are written
    - Complete all items
    - Make sure the response you get actually answers the question asked
    - Get clarification whenever you are uncertain how to complete a form or code an answer
    - Make sure there are no missing data on the form
    - Record time using a 24-hour clock from 00:00 (midnight) to 23:59. DO NOT RECORD 24:00.

## Scanning Completed CRFs

The RA should scan all circled items on the checklist & progress note once the CRFs are completed, signed (if required), and reviewed.

Instructions for scanning documents (use LANIER copy machine):

1. Place document(s) face-up in the printer
2. Press the *scanner* button
3. Enter the study copy code
4. Touch the *store file* button on the screen
5. Touch the *store only* button on the screen
6. Touch the *OK* button on the screen
7. Press the green *START* button
8. Stamp each page that has been scanned on the reverse side

## Source Documents

Source documents for a given piece of information consist of the first place that the information was recorded. This can be a progress note, laboratory report, a phone message, or the napkin that you wrote a phone message on. Source documents include all notations of clinical activities and all reports and records necessary for the evaluation and reconstruction of the clinical research study. Accordingly, source documents include, but are not limited to, laboratory reports, ECG tracings, X- rays, radiologist reports, subject diaries, biopsy reports, ultrasound photographs, subject progress notes, hospital charts or pharmacy records, and any other similar reports or records of any procedure performed in accordance with the protocol. Whenever possible, the original recording of an observation should be retained as the source document; however, a photocopy is acceptable provided that it is a clear, legible, and exact duplication of the original document.

## Progress Notes

Progress notes are very important to protocol documentation. Progress notes should tell what happened, what didn’t happen that should have, and plans for the next visit. Progress notes should be concise, professional, informative, signed and dated, and should NOT contain the participant’s name. Information in progress notes can be both clinical and research in nature and may include a summary of your interaction with the participant, as well as documentation of data collection. Progress notes must be completed for every visit with a participant, as well as for missed visits, and should be kept in the Participant Data File. A checklist of required procedures that should occur during the visit accompanies the visit-by-visit progress note document. The checklists serve as a reminder and confirmation that study procedures were completed according to the protocol.

# IMPORTANT RULES TO REMEMBER ABOUT PROGRESS NOTES:

- - - Always write a progress note for each visit of the study. Good Clinical Practice encourages that the note is written immediately after the visit.
    - Always include the date
    - Include notes about
      - What happened.
      - What did not happen that should have.
      - Notable information about participant’s involvement (e.g., level of involvement in treatment, willingness/capacity to complete study-specific measures, etc.).
      - Plans for next time, if any.
      - Other contacts with participant (e.g., phone calls, letters, etc.) to follow up on missed appointments, schedule research or follow-up appointments, and contacts made at the request of the participant with release of information (e.g., primary care physician or other medical specialist).
    - Limit use of abbreviations.
    - Always sign and date your progress notes.
    - List items (e.g., data collection activities) in the order that they occurred because Quality Assurance monitors will assume that this is the order in which they were administered. This is critical when documenting the informed consent process.
    - Pay special attention to documentation of safety issues for participants (e.g., assessment for suicidal ideation).
    - Write enough information to document the activity or data, but don’t go overboard. Notes should be brief and to the point.
    - Avoid using names in the progress notes (e.g., refer to “participant” rather than “Mr. Jones”).
    - If you need to refer to another person, refer to them by relationship rather than by name (e.g., “participant’s girlfriend”, not “Betty Smith”).
    - Don’t leave blank spaces or blank lines between notes. If you do leave a blank line for some reason, draw a line through it. This prevents the appearance that space is being left in case you need to add more information later. Also, fill in the last line of the note with a line to the end of the page.
    - If you were unable to complete documentation on the date that a research or treatment activity occurred, write a late note. Never backdate. Backdating is putting a date other than today’s date on a form or note (e.g., putting yesterday’s date on a note because that is the day that the data collection occurred). This is a form of data falsification.

## Study Logs

Study logs are used to document study-related activities. This study requires the use of the following logs:

- - - Pre-Screening Log
    - Master Enrollment Log
    - Gift Card Log
    - Signature and Responsibilities Log
    - Site Visitor Log
    - Protocol Violations Log

Templates for required study logs can be found in the appendix.

## Master Enrollment Log

The Master Enrollment Log will list all participants who have signed consent to participate in this study and will track their status through randomization. This log will document the participant ID number and name code, date of consent, date of induction, date of randomization, and randomization group. This should be the ONLY document that links the participant ID# and the participant’s name,

and must be kept in a locked file cabinet. Only authorized research staff should have access to this Log.

## Document Storage

Study materials should be filed promptly and securely to protect the participant’s identity. Guidelines for assembling the regulatory files and participant files can be found in Sections 2.4 and

2.5 of this manual. Government agency regulations and directives require that the investigator must retain all study documentation (data corrections forms, workbooks, CRFs, source documents, monitoring logs and appointment schedules, correspondence and regulatory documents) pertaining to the conduct of a clinical trial. Additional government requirements for retaining study documentation specify that treatment research data be kept for a period of at least seven years. Thus all study documents will be kept for a minimum of seven years before destroying.

## REMEMBER: STUDY DOCUMENTATION MUST NEVER BE DESTROYED, UNDER ANY CIRCUMSTANCE!!!

Even if a document is no longer being utilized, or is seemingly illegible due to multiple error corrections, do not destroy or get rid of it.

## 4.0 Study Interventions

After consent, participants will be administered the MINI and DSM-IV checklist, the shortcode test message will be tried and adherence calculated. If eligible, they will be randomized to either the TXT-CBT condition or the pamphlet condition.

Participants for both conditions will then be administered baseline assessments and be coached for unannounced pill counts by the research assistant. Participants should be instructed on how to read the labels of their pill bottles (Rx number, number of pills, name of medication, date dispensed, and number of refills left). At baseline, participants will also be asked to either provide a copy of their latest CD4/Viral load count or to sign a medical release form that allows the RA to contact their medical provider in order to get their CD4/Viral load count for the duration of the study.

Participants randomized to the TXT-CBT group will then meet with a CBT counselor for a briefing on life steps. At this time, the CBT counselor will fill out the life step form and give it to the RA who will enter the data on the electronic form.

Upon completion of this session, the RA will go over the Participant Manual with the participant and fill out the Tailoring Variables form that is to be faxed to Santech as soon as the participant leaves. At the end of the baseline visit, an appointment for the participant’s 4th week should be made and the RA should remind the participant to expect a phone call for pill count at some point during the month. For weeks 4, 8, and 12 participants will be scheduled to come in the clinic to fill out their monthly assessment, provide a urine sample and trouble shoot any technical problems with the RA. At the end of each visit, an appointment for the participant’s next visit should be made and the RA should remind

the participant to expect a phone call for pill count at some point during the month.

## Study Research Procedures

## Screening and Baseline Assessments (Week 0)

Screening assessments, comprising of biopsychosocial measures (with RA staff) are intended to ensure that potential participants meet all eligibility criteria before being randomized.

# The informed consent MUST be signed before ANY screening assessments are begun.

The RA will review the consent form with participants.

The following table shows the staffing assignments for all of the procedures to be completed during the Screening and Baseline Assessment phase. Study staff should make every effort to complete the set of screening/baseline assessments in one visit; however if necessary, the assessments may be completed in two visits.

## Table 5.1 Screening and Baseline Visit Procedures (Week 0)

| **Procedures** | **Administered/Collected By** |
| --- | --- |
| Informed Consent | RA |
| Mini International Neuropsychiatric Interview (MINI) | RA |
| Inclusion/Exclusion | RA is responsible for determining whether a participant is eligible. This should be noted in the Screening Progress Note. |
| Participant Compensation Receipt | RA |
| Schedule Next Clinic Visit | RA |
| Test Text message to determine short code capability (Tell participant to text in ‘test’ to 843438) | RA |
| Checklist & Progress Note | RA |

## Screening Visit

Based on the table above, the RA will be responsible for collecting the biosychosocial measures with prospective participants. The substance dependence diagnosis and items from the Inclusion/Exclusion Criteria lists will be determined from these measures.

# Informed Consent

The informed consent form must be reviewed and signed before any biopsychosocial screening assessments are done. The purpose of the informed consent form is to educate potential participants about the study procedures, including eligibility, randomization, treatment types, duration of study, and all study procedures conducted by the RA and medical staff. Those who consent to participation will receive the screening assessments and, if eligible, begin the intervention.

In handling all informed consent procedures, always make sure that the consent form is the current, IRB-approved version. Forms are required to have an IRB stamp of approval, dates, and/or signatures. It is the research staff’s responsibility to understand how consent form versions are tracked. Only the current, IRB-approved version of the consent is valid. If a participant is accidentally given an older version of the consent and signs it, the participant will need to review and sign the current approved version as soon as possible. The research staff must ensure that the participant has understood the consent form by reviewing it with the participant, and then obtaining the participant’s signature. Consent form errors such as using outdated versions or forgetting to get a signature are the most common type of protocol violation.

To avoid making these errors, do the following when obtaining informed consent:

- - - - - Introduce yourself to participant and explain your role in the study
        - Introduce the study to participant
        - Give a brief description of the purpose of the consent process, including the amount of time involved
        - Summarize the study (purpose, procedures, visit schedule, risks/benefits, compensation, alternatives)
        - Explain the confidentiality assurances, including the Certificate of Confidentiality, as well as limitations
        - Determine if participant would like to review the consent alone, with another person, at home, or is ready to review the consent with you. The Investigator or other research staff must ensure that the participant is provided opportunity to consider whether or not to participate
        - Highlight the participant’s rights, including the voluntary nature of the study, the right to withdraw at any time, and the contact names and phone numbers
        - Have participant sign the consent form and date it (check to make sure this is done as participants often forget to date)
        - Remember to have the Investigator sign and date the form
        - Give a copy of the signed consent to participant
        - File original signed consent form in a secure location and a copy in participant’s name file
        - Thank participant for being in the study

An important aspect of the consent procedure is not only that the participant is informed of the procedures, but also that the participant is aware of what is required of him/her to participate. This includes understanding how many visits he/she must attend, how many urine drug screens are scheduled, and how many assessment visits he/she will be asked to complete.

# Collection of Locator Information

Follow up is critical in any research protocol. By tracking participants across time and comparing outcomes at termination to outcomes at follow-up points, documentation of stimulant use over time will be possible. The Locator Form is the primary way to obtain and maintain contact information for the participant. The participant will be asked to fill out the Locator Form and then the RA will review the form with him/her in order to fill in the blanks and obtain complete information. It should be completed at screening and updated weekly. Careful and detailed use of the Locator Form and other retention strategies is the only way to get sufficient follow-up for useful information. The Locator Form is contained in the appendix.

# Assigning Participant ID Number

Each participant who has provided informed consent should be assigned a participant ID number. The participant ID number will consist of four digits and will be assigned in sequential number. The first consented participant should be assigned the number 7000, the second should be assigned 7001, and so on. The participant will keep the same participant ID number throughout the study, and the number will serve as his/her confidential identification on study forms. The participant ID is a unique identifier for each participant and should not be duplicated or reassigned to another participant. The participant ID number will be recorded on the Master Enrollment Log (see Appendix).

## Confirming Study Eligibility

The prospective participant should not be enrolled into the study until all of the results from the screening assessments reviewed.

# Inclusion Criteria

1. Age 18 or older;
2. DSM-IV diagnosis of Alcohol Dependence;
3. Use of 5 or more standard drinks per drinking day for men or 4 or more for women (over the past 30 days);
4. HIV-infected serostatus;
5. Able to provide informed consent;
6. Willing and able to participate in study procedures,
7. Good general health or, in the case of a medical/psychiatric condition needing ongoing treatment, potential participant should be under the care of a physician who provides documented willingness to continue participant’s medical management and coordinate care with the study physicians.
8. Adherent to <90% of antiretroviral therapy regimen, as determined by our medication adherence screening procedure
9. Owning a cell phone. Participants are required to own their phone and cannot use someone else's phone.

# Exclusion Criteria

1. Presence of serious medical condition that would, in the opinion of the study physician, make participation medically hazardous (e.g., acute hepatitis, unstable cardiovascular, liver or renal disease).
2. A current pattern of alcohol or sedative use, as assessed by the study physician, which would preclude safe participation in the study and/or would likely require imminent medical detoxification.
3. Has undergone more than one inpatient medical detoxification treatment;
4. Lack of proficiency in English;
5. Currently homeless (unless residing in a recovery home for which contact information can be provided);
6. Presence of clinically significant psychiatric symptoms as assessed by MINI, such as psychosis, acute mania, or suicide risk that would require immediate treatment or make study compliance difficult.
7. Adherent to > or =90% of antiretroviral therapy regimen, as determined by our medication adherence screening procedure.

## Handling Failed Screeners

Some participants will not meet all eligibility criteria for the study, or may be eligible but decide not to participate. In all cases, such a participant will be considered a screen failure. Failed screens will not be enrolled into the Compliance Phase or randomized/inducted onto study medication. Instead, they should be referred to a local treatment facility and/or other research opportunities (the latter if requested). Once it is determined that a participant is ineligible, the remaining eligibility assessments do not need to be completed. **However, ALL screened participants must have at least the Demographics, Inclusion/Exclusion, and Study Termination CRFs completed, as well as the reason for exclusion documented on the Baseline/Screening Assessment progress note.** Progress notes for failed screeners should reflect the procedures completed before the participant was ruled out, and any other relevant information on the participant’s ineligibility.

## Scheduling

The RA should make an effort to schedule the participant’s next appointment in person during his/her visit. All scheduled appointments must be added to the Clinic Research Center Appointments Calendar (OCRC). This calendar can be accessed through the internal ISAP webpage. Each appointment should be entered as follows: study number (10) - Participant name (week # visit #). For example is participant Jane Doe was in her 2nd week 1st visit, her appointment would be entered as: 10

- Jane D. (Week 2 V.1).

## Baseline Assessments

| **Procedures** | **Administered/Collected By** |
| --- | --- |
| Participant Locator Form | Participant self-report /RA |
| MINI | RA |
| DSM-IV Checklist | RA |
| PHQ9 | RA |
| OASIS | RA |
| Reback Behavioral Risk Assessment | RA |
| Subjective Effects of Alcohol Scale | RA |
| Breathalyzer | RA |
| Urine Drug Screen | RA |
| Penn Alcohol Craving Scale | RA |
| Addiction Severity Index Lite CF (ASI) | RA |
| Timeline Follow Back (TLFB) | RA |
| Medical Marijuana | RA |
| Socrates A | RA |
| Viral Load | Participant self-report/Labs |
| Health Status Questionnaire (SF-12) | RA |
| Drug Confidence | RA |
| Self-Efficacy | RA |
| Vivitrol Questions | RA |
| Pill count/Adherence | Participant self-report/RA |
| Medication compliance | RA |
| Behavioral Strategies | RA |
| Participant Compensation Receipt | RA |
| Concomitant Medications | RA |
| Ancillary Treatments | RA |
| Schedule Next Clinic Visit | RA |
| Checklist & Progress Note | RA |

If the participant withdraws or is terminated early from the study at any time, the RA should complete the Study Termination Form and provide local treatment referrals and medical referral for any open Adverse Events as needed.

## Random Assignment

As noted in the protocol, participants will be randomized to treatment condition. The randomization procedures should be completed as soon as the participant’s eligibility is determined.

Randomization Procedures

To randomize a participant, log in to the DMC website <http://www.uclaisap.org/dmc/>and click on “randomize a participant”, enter the requested information to randomize the participant. Once the participant has been randomized, enter their treatment condition into the Master log. Also give the participant the appropriate congratulatory letter (see appendix).

## Phase 3: Intervention (Weeks 4, 8 and 12)

During the intervention phase participants will attend the clinic monthly (once a month) for collection of data and urine specimens.

| **Procedures** | **Administered/Collected By** |
| --- | --- |
| PHQ9 | RA |
| OASIS | RA |
| **Reback Behavioral Risk Assessment | RA |
| Subjective Effects of Alcohol Scale | RA |
| Breathalyzer | RA |
| Urine Drug Screen | RA |
| Penn Alcohol Craving Scale | RA |
| **Addiction Severity Index Lite CF (ASI) | RA |
| Timeline Follow Back (TLFB) | RA |
| **Socrates A | RA |
| Viral Load | Participant self-report/Labs |
| **Health Status Questionnaire (SF-12) | RA |
| **Drug Confidence | RA |
| Self-Efficacy | RA |
| Vivitrol Questions | RA |
| Pill count/Adherence | Participant self-report/RA |
| Medication compliance | RA |
| **Behavioral Strategies | RA |
| Participant Compensation Receipt | RA |
| Concomitant Medications | RA |
| Ancillary Treatments | RA |
| Schedule Next Clinic Visit | RA |
| Checklist & Progress Note | RA |

** Only at week 12

If the participant withdraws or is terminated early from the study at any time, the RA should complete the Study Termination Form and provide local treatment referrals and medical referral for any open Adverse Events as needed.

## Missed Visits

Every effort should be made to reach participants within their visit window if they miss their scheduled visit for some reason. For this reason, it’s always a good idea to schedule participants earlier in their visit window than later, in case a visit needs to be re-scheduled. The main outcome measure for this study is methamphetamine use, so getting the urine drug screen from participants at every scheduled visit is especially important.

If a participant misses their scheduled appointment time, research staff will attempt to contact that participant within a half hour of the original appointment time. If research staff are unable to make contact with the participant that day to reschedule the appointment, they will continue to contact that participant once a day via telephone, email, or standard mail for a period of two weeks.

## Biological Assessments

- 1. **Urine Drug Screens How does testing work?**

Urine drug tests do not test for the presence of the actual drug. When the body breaks down (metabolizes) the drug, certain by-products (metabolites) are left behind. These are removed from the body by the kidneys and exit the body in the urine. Drug tests evaluate the urine for the presence of these by-products. Each drug has specific metabolites associated with it. Therefore, by testing the urine for the presence of metabolites associated with each of the drugs under investigation, an assessment of recent use can be made. Urine tests are only as valid as the collection procedures used. There are ways of cheating a urine drug test. These include diluting the sample, putting chemical agents in the urine (e.g. bleach) or bringing in someone else’s urine. Understanding and using appropriate monitoring methods is critical to ensure accurate testing.

## Windows of Time for Drug Detection

The amount of time that it takes for the body to remove the drug from the body (and therefore the amount of time that someone will test positive following the last use of the drug) differs for each substance. While the exact amount of time varies by person and the amount of drug used, some rough guidelines are presented below:

*Cocaine and Methamphetamine***:** These drugs are broken down fairly quickly and remain detectable for about three days.

*Opiates****:*** Opiates take a little longer for the body to process. These drugs are still detectable for three to five days after the last use.

*Marijuana****:*** This drug is stored in fat and is, therefore, detectable for a much longer period of time. Marijuana is detectable in the urine for as long as thirty days.

*Benzodiazepines:* These drugs are detectable in the urine for up to thirty days.

## Procedure for Drug Screening

**Note: Staff should observe the temperature strip on the collection cup immediately after the participant has provided the sample to guard against tampering, but research staff do NOT need to observe the participant urinating.**

All drug screens will be collected using temperature controlled urine test cups. For this measure, an FDA-approved one-step test will be used. Following collection of the urine sample, staff will immediately perform the test, wait for the results and record the results on the appropriate study form. Instructions on how to use the test must be followed carefully in order to obtain accurate results.

This study will use a 6-panel, one-step, “dip and read” urine test card for the qualitative detection of the following drugs and drug metabolites in human urine:

Opiates (OPI) Benzodiazepines (BZO) Cocaine (COC)

Amphetamines (AMP) Methamphetamines (mAMP) Marijuana (THC)

*Storage and Stability*: The test card should be refrigerated or stored at room temperature 2 o-30o C (36 o- 86oF) in the sealed pouch until time of use. DO NOT FREEZE. Do not use the test card beyond the date printed on the sealed pouch. If refrigerated, the test card should be brought to room temperature (15o- 30oC/59 - 86oF) prior to testing.

*Safety*: Be aware of safety and hygiene when handling urine. Staff should wear latex or polyurethane gloves, lab coat, safety glasses and clean the area regularly with 1 part bleach and 9 parts water.

*Urine Collection*: Use fresh urine specimens. Urine specimens do not require any special handling or pretreatment. **Allow the urine specimen to equilibrate to room temperature (15o- 30oC/59 - 86oF) prior to testing.** Handle and dispose of urine specimens as if they were infectious and capable of transmitting infection.

- - 1. Label the urine container with the participant’s ID # and collection date before sample is collected. Write the information in waterproof ink.
    2. Give the participant the designated sample cup in which to urinate. Be sure that the temperature cards are secured in the bottom of the cup.
    3. Inspect the urine for possible tampering if a sample is deemed suspicious, collect a second sample and note what happened on the visit progress note.
    4. Label the test device with the participant’s ID # and collection date before starting test. Write the information in waterproof ink.
    5. Remove the test card from the sealed pouch and use it as soon as possible.
    6. Remove the cap from the end of the test card to expose the absorbent tips.
    7. Dip the strips of the test card in the urine specimen to at least the level of the wavy lines on the strips. Do not allow urine to touch the plastic portion of the test card when immersing the strips. Keep the strips in the urine for at least 10-15 seconds.
    8. Replace the cap to cover the strips. Place the test card on a flat surface.
    9. Results of the custom one step drug screen test card should be read at 5 minutes. **Results remain stable for up to 4 hours.** Record results on the appropriate CRF.
    10. If for any reason you do not read the results within the specified timeframe, the test is not valid. You must repeat the test using a new test card rather than recording results obtained outside the testing window.

*Urine Disposal:* Handle and dispose of urine specimens as if they were infectious and capable of transmitting infection. Once testing has been completed and verified, study staff should dispose of the urine sample in the specified sinks.

## Validity Checks for Urines

*Visual Inspection of the Sample*: One important way of monitoring the sample is to look at it. The sample should look like urine. A sample that is too clear may have been diluted as a way of reducing the amount of drug metabolite in the urine. In cases where the urine sample appears to be diluted or tampering may have occurred, a second sample should be requested as soon as possible.

*Odor:* Participants may attempt to adulterate their samples with bleach. If a sample has a distinct chlorine, swimming pool, or bleach smell, it should be considered suspect. In cases where the urine sample smells like it has been tampered with, a second sample should be requested as soon as possible. Staff are NOT instructed to smell the urine; rather, staff are given the guideline that if they happen to notice this sort of odor, they are not to ignore it.

*Temperature:* Urine that has just come from the body should be approximately body temperature. The temperature cards record the temperature as soon as it is collected. Samples that register below 96 or above 100 degrees Fahrenheit should be considered suspect. In cases where the urine sample is too hot or too cold, a second sample should be requested as soon as possible.

## Interpretation of Results

- *Negative:* Two colored lines adjacent to each drug name should be observed in the viewing windows. The line in the test region (T) is the drug probe line; the line in the control region

(C) is the control line, which is used to indicate proper performance of the device. The test line intensity may be weaker or stronger than that of the control line.

- *Positive:* Only one colored line appears in the control region (C). The absence of a test line indicates a positive result for that drug.
- *Invalid:* No line appears in the control region (C). Under no circumstances should a positive sample be identified until the control line (C) forms in the viewing area. If the control line (C)

does not form, the test result is inconclusive and the assay should be repeated with a new test card.

## Urine Drug Screens

**On-Site Alcohol Breathe Testing**

Alcohol use by participants is tested during each study visit. A hand-held breath alcohol tester will be used to measure the subject’s breath/blood alcohol level. During each study visit, the subject will be asked to provide a breath sample to measure the level of alcohol content in their blood. The device consists of two pieces; a disposable mouth piece and measurement unit.

***Process for taking a sample:*** Remove cover from mouth piece Place mouth piece into slot provided

Ask the participant to take a deep breath and blow into end of mouthpiece for at least 3 seconds. A negative response will display within 5 seconds

A positive response will display within 30-45 seconds Press the eject button on analyzer to remove mouthpiece

The results of the testing will be displayed as a three character numeric and entered onto the “LDN Urine Drug Screen and Breathalyzer Results” CRF. If no reading is displayed then a new mouthpiece should be used and test repeated.

Calibration procedures will be performed on the breathalyzer machine on a regular basis according to the manufacturer’s specifications.

## Participant Discontinuation/Termination

## Criteria for Termination

The participant or the investigator can initiate study discontinuation. Participants may withdraw from the study at any time. There are no consequences for voluntarily withdrawn consent.

The investigator may terminate a participant if s/he deems it clinically appropriate or for any reason, including the following:

- - 1. Serious or unexpected AEs
    2. Inability to comply with the study protocol
    3. Protocol violation
    4. Missed 1 month of data collection

## 7.1.1 Participant Incarceration

Contact or follow-up of participants during the time in which they may be considered “prisoners” under 45CFR46.303(c) may only be made with IRB and OHRP approval to do so. If there is not IRB and OHRP approval to contact prisoners, an attempt will be made to follow-up such participants once they are no longer considered prisoners unless they are released more than 49 weeks following the date of their randomization.

If the participant misses more than 4 consecutive weeks of study visits after randomization as a result of their incarceration the participant will be terminated from the study. Once the participant is released, every effort should be made to make contact and complete termination assessments as specified in the protocol. If the participant is released before missing 3 consecutive weeks of study visits after randomization, they may be re-inducted and continue in the study.

## 7.2 Procedures for Discontinuation

If a participant is withdrawn from the study, s/he should not be re-admitted. Any un-captured data for missed visits beyond the date of study discontinuation will be coded as missing. Research staff will complete the Study Termination Form and make sure to note the reason in the progress note.

Participants who complete the study (do not terminate early) through Week 24 should also have a Study Termination Report completed on the appropriate CRF. Their study completion will be documented on the Week 16 checklist & progress note.

Note: Only one Study Termination Report should be filled out for each participant.

## Recruitment and Follow-up Topics

## Recruitment

Recruitment activities will continue until the target number of participants (N = 50) has been randomized into treatment conditions (2 conditions with n = 25 each). We expect that approximately 75 alcohol dependent adults with HIV-infection seeking treatment will be screened for participation in this proposed project.

Participants will be recruited through advertising, word of mouth, study announcement fliers posted in treatment programs and community locations, referrals from local substance abuse treatment and outreach programs, outpatient and inpatient alcohol and drug abuse clinics, primary care providers, local mental health centers, crisis clinics, public service announcements, hospital emergency rooms, and self-referral. The sample will be stratified by gender. All recruitment materials refer interested persons to clinic phone numbers, which are answered by a research staff member trained to provide the caller with information about the study and to schedule interested persons for an in-person interview.

## Retention Techniques

Good retention is the key to most efficiently reaching the target sample size and providing the data needed for analysis. Study participants will be required to come to the clinic once a month and will be called for an unannounced pill count once a month as well. This provides a number of opportunities for research staff to maintain participant contact and ensure that participants meet with staff in order to complete scheduled assessments. It is vitally important that good rapport with participants be maintained so that the research assessments—especially safety data—can be collected consistently, without participants feeling that those assessments are burdensome and not worth completing. The research staff will coordinate availability so that it is as easy as possible for participants to complete their study visits at times that are convenient to them.

During screening the participant will be asked to fill out the Locator Form and then the RA will review the form with him/her in order to fill in the blanks and obtain complete information. Even with the best of intentions, some participants may slip through the cracks and the RA will need to work extra hard to retain them in the study. This is where current locator information and persistence come into play. Many times, participants are very hard to contact and progressively more difficult to stay in touch with over the course of the study. It is vital that you keep each participant’s Locator Form updated with correct and current contact information. This will greatly improve research staff’s

ability to track participants for ongoing visits. It usually takes multiple attempts to contact participants and it is an even harder task to schedule appointments. These measures will be taken to increase retention and make the research staff, specifically RAs, more confident in their abilities to keep participants coming back.

- - - The space where research interviews are conducted will be comfortable and staff will be accommodating to reasonable requests of participants (i.e., refreshments are available, the space is neither too hot nor too cold).
    - Study staff will work diligently during the active phases of the study in order to develop rapport with the participants.
    - All attempts will be made to contact participants in advance of the date that their assessments are scheduled and to work with them to secure the desired date of the appointment.
    - Many strategies will be used to make contact and locator information will be updated at each visit. As long as all attempts are ethical, there will probably be a greater outcome than if only one method of contact is used.
    - Participants will be reminded of compensation for time and effort due for a given visit. This is one of the benefits they receive for participating and it might help to create incentive for keeping appointments.
    - Study staff will maintain a positive attitude and try not to get discouraged. They will keep high expectations and look to achieve attendance at each scheduled visit. They will keep trying and be persistent.

## Lost to Follow-up

Follow-up efforts include letters, telephone calls, and, if possible, email contact with the study participant as well as with other contacts listed on the study Locator Form (i.e., friends, family, roommates, and employers).

## Data, Safety, and Monitoring

## Adverse Events (AEs)

An Adverse Event (AE) is defined as any reaction, side effect or untoward event that occurs during the course of the clinical trial, whether or not the event is considered investigational agent related or clinically significant. For this study, AEs include events reported by the participant, as well as clinically significant abnormal findings on physical examination or laboratory evaluation. A new illness, symptom, sign, or clinically significant laboratory abnormality or worsening in frequency or severity of a preexisting condition or abnormality that occurs during the course of the study is considered an AE. Stable chronic conditions, such as arthritis, which are present prior to clinical trial entry and do not worsen are not considered AEs. Withdrawal symptoms will be recorded as such on the AE/SAE CRF.

Any adverse experience will be noted in the adverse events section of the participant’s research chart. The study physician or designee will evaluate the intensity, seriousness, and causal relationship to the study intervention of all adverse experiences, whether or not thought to be related. Each visit the study physician or designee must review the AE/SAE CRFs completed for the previous visit for any events that were reported as continuing. All AEs, including clinically significant abnormal findings on laboratory evaluations, regardless of severity, will be followed until satisfactory resolution. Continuing AEs will be reported up to 4 weeks following completion of, or termination from treatment.

## Serious Adverse Event (SAEs)

Each adverse event or reaction will be classified by the study physician or medical personnel as serious or non-serious. Based on the seriousness of the adverse event or reaction, appropriate reporting procedures must be followed

## Confidentiality

The research team is responsible for ensuring that the confidentiality of the participant is protected, to the extent permitted by applicable laws and regulations, throughout the study and after the individual’s participation in the research study has ended. These expectations are mandated in federal regulations governing confidentiality.

## Certificate of Confidentiality

A Certificate of Confidentiality specifically protects the privacy of individuals participating in alcohol and drug abuse research studies. Under the Public Health Service Act, any researcher who engages in studies of alcohol and drug abuse may obtain a Certificate of Confidentiality. A researcher who has obtained a Certificate of Confidentiality cannot be compelled to identify research participants in any Federal, state, or local civil, criminal, administrative, legislative, or other proceeding. A copy of the certificate is filed in the Regulatory Binder.

Certificate of Confidentiality Link: <http://grants.nih.gov/grants/policy/coc/index.htm>

## Breach of Confidentiality

An accidental breach of confidentiality can occur when:

- The identity of a study participant is made known (except when given permission to do so by the participant).
- Information that could link the participant with activities conducted by the study is made known. Examples are:
  - identifying information on correspondence
  - telephone numbers linked to treatment facilities
  - leaving identifying messages
- Information that could be linked to a particular individual is made known. Examples are:
  - an address
  - information about some criminal act or other matter that may be documented in the public record
- Identifying information is included in the case history.

Do your best to think through your response before answering any question about participants or their involvement in the study from anyone not directly involved with the study.

If a breach of confidentiality occurs, document the event in a “Note to File” and notify your Principal Investigator and IRB, as required.

## Maintaining Confidentiality

The research team is responsible for protecting the confidentiality of information obtained from the participants. It is important to work as hard as possible to maintain the participant's privacy. This should be done in the following ways:

- Never let confidential information out of your control by leaving it lying out on your desk, leaving it in a printer, FAX, or unattended computer, or traveling with it in your car, etc.
- Never record the participant's name on data forms.
- Always use the participant's ID number on questionnaires and other data forms.
- Identifying information (e.g., names, social security number, address, phone number, etc.) should be kept in a locked file or secured office. If there is a need to link this information, it can be done by keeping a log of participant names and ID numbers. This log must then be kept in a locked file.
- To ensure the participant's safety as well as for study follow-up, names and phone numbers of contact persons should be obtained and placed in a locked file that is only accessible to those who need to have the information for study purposes. Permission to collect this information for the purpose of follow-up must be given by the study participant and be noted in a progress note.
- Keep control of data records by having a system (e.g., binders) for keeping each participant's information organized. Keep this system up-to-date by filing data promptly. This helps to ensure that important pieces of data are not accidentally missed and prevents information from being lost or seen by someone not working on the project.
- Discuss participants only with other study personnel. Do not talk about participants while in public areas such as hallways or elevators. Do not talk about participants to employees who are not part of the study.
- Make sure a release-of-information form has been obtained from the participant if information is provided to non-study related sources.

In general, if inquiries are made as to whether a particular person is participating in the study, you do not have the right to confirm or deny the individual’s participation. If someone calls asking to talk to a participant or inquiring about a participant, one might respond by saying: *"Participation in this study is confidential. I cannot confirm or deny that this person is enrolled in this study”.*

## Exceptions to Confidentiality

Not all information provided by the participant is confidential. Participants may voluntarily disclose information that is required by law to be reported to the authorities. Participants should be informed of these exceptions to confidentiality prior to enrolling in the study.

Information about a participant can be disclosed:

- To people performing duties related to the patient’s diagnosis, treatment, or referral for treatment of alcohol or drug abuse
- To law enforcement officers when the patient has committed, or threatened to commit, a crime on program premises or against program staff
- When reporting suspected child abuse or neglect to state or local authorities
- To medical personnel in a medical emergency
- For management audits, financial audits, or program evaluation
- If a patient is found to be at risk for suicide or if he or she makes a credible threat to harm another person
- When the patient has a communicable disease that poses a risk to public health
- When required by state law

If in doubt about disclosing the requested information, consult the regulatory staff.

## Release of Information

Title 42 of the Code of Federal Regulations (CFR) Part 2 covers “confidentiality of alcohol and drug abuse patient records” and indicates that programs may not use or disclose any information about a participant unless the participant has consented in writing (on a release of information form that meets the requirements established by the regulations) or unless another very limited exception specified in the regulations applies. Any disclosure must be limited to the information necessary to carry out the purpose of the disclosure.

Without a release of information, the study staff will be unable to provide ANY information to the participant’s significant others including whether or not the candidate is participating in the study. If the participant has requested that no information be left with another person or on an answering machine, then a message should not be left.

## Emergencies

The study site will need to follow the established, site-specific practices for managing medical and psychiatric emergencies. The procedures are documented as a Standard Operating Procedure (SOP) and are filed in the Standard Operating Procedures section of the study regulatory binder. All research staff should review and understand the established emergency procedures prior to implementing the study.

## Suicidal Participants

If any member of the study team encounters a participant who expresses suicidal intentions or suspects that a participant may be suicidal, the following steps should be taken:

1. Assess whether the participant is suicidal:
   - Ask him/her directly (Are you thinking of killing yourself?) Do not use euphemisms (Are you thinking of hurting yourself?)
   - Follow-up on off-handed remarks (i.e., participant mentions desire to “stop the world and get off”). Do not assume that joking manner means the words are a joke.
2. Once participant’s suicidal ideation is clear, assess the likelihood of an attempt:
   - Ask whether the participant has plans or the means to carry out a suicide attempt because it provides an indication of seriousness.
   - Assess whether the participant has a history of suicide attempts. (The best predictor of future behavior is past behavior.)
   - Assess whether the participant has a support group.
3. Conduct **SLAP** assessment:
   - **Seriousness/Severity** of ideation (low or high seriousness/severity – does s/he have a plan etc.)
   - **Lethality** of chosen means (how lethal is their chosen plan – shoot with a gun, overdose on drug of choice, Tylenol)
   - **Accessibility** of chosen means (does s/he have the gun, drugs, etc. available)
   - **Proximity** of social support (family, friends available/near to lean on)

Once the mentioned assessments have been conducted, the participant should meet with the supervising counselor (or physician/medical personnel if the counselor is not available), who will need to take the following steps where appropriate:

1. Determine the participant’s suicide risk
2. Determine mitigating factors (reasons participant will not act on his/her suicidality – loss to others, religion, etc.)
3. Develop a contract with the participant if it is determined that s/he has the ability to make such an agreement:
   - Suicide contract – attempt to contract with participant an agreement that s/he will engage in a number of itemized activities rather than attempting to carry out his/her suicide plan. The participant should write out the contract (or the counselor can write it out if necessary) based on the particulars of the agreement. Items differ based on the participant and his/her circumstances, but can include: participating in other activities, talking with family or friends in the immediate future for support, attending a 12-Step meeting, calling his/her sponsor, calling the suicide hotline (check number first) or 911, going to a hospital for help
4. Contact support group:
   - Counselor could ask the participant to call family/friends in his/her presence
5. Provide referral information:
   - Give the participant a list of at least 3 referrals for mental health treatment and a suicide or emergency hotline. Study staff should call the numbers on the list to ensure they are valid before giving them out to a participant.
6. Determine whether the participant can be dismissed after the interview, or whether authorities should be contacted:
   - If appropriate, the counselor should instruct the participant that they are in imminent danger and need to go for immediate help. S/he can be instructed to go to an emergency or psychiatric hospital and report the intent to commit suicide. This will result in being admitted.
   - If the participant will not agree to contract, call the Psychiatric Emergency Team (PET) or the police if PET is unavailable. If the participant agrees to this intervention, the counselor should make the call in his/her presence. If it seems the participant will not agree or will be hostile about calling for intervention, the counselor should make the call surreptitiously. If the counselor has reason to believe s/he will be threatened for making the call, the call should be made after the participant leaves.
7. If the police respond to a 911 call for assistance:
   - Police will “secure the scene,” which will likely involve handcuffing the participant (if appropriate, inform the participant of this so s/he is prepared)
   - Police will want to interview the participant and may ask the counselor to leave the room. **Ask the participant if s/he wants the counselor to leave the room and act accordingly**.
   - Police will ask the participant about wanting to kill himself/herself, but may pose the question in such a way that the participant is coerced into saying s/he does not intend to commit suicide. The counselor should be prepared for this, especially if s/he is with the participant during the police interview.
   - If the police release the participant based in the interview, make sure the participant has emergency referral information when s/he leaves.
8. If the participant leaves before 911 assistance arrives, the counselor should give the police all contact information and specifics about the participant’s direction, etc.

## Staff members must do everything possible to dissuade the participant from suicide and steer him/her towards help, but the bottom line is that study staff is not responsible for whether the client ultimately succeeds in killing him/herself.

**Issues of Confidentiality**

Study staff is mandated to protect the health and welfare of the participants even if it means breaching confidentiality. Although it may be necessary to report the participant’s name and address to authorities in order to obtain assistance, every other piece of information about the participant not related to his/her suicidality is to remain confidential. This means study staff must maintain confidentiality in regards to participant on the study, drug treatment, drug use history, etc.

## Homicidal Participants

If any member of the study team encounters a participant who expresses homicidal intentions or suspects that a participant may be homicidal, the same protocol for suicidal participants should be followed (including the SLAP assessment).

If the participant is imminently homicidal, study staff has a “duty to warn:”

1. Call the police
2. Notify the intended victim

(Note: Do not make these calls with the participant present.)

## Issues of Confidentiality

With homicidal participants, study staff is mandated to breach confidentiality to protect the intended victim’s health and safety. Staff is not given permission to break confidentiality in regards to the individual’s participation in this study, drug treatment, drug use history, etc.

## Reporting Child/Elder/Dependent Adult Abuse

UCLA employees are legally mandated to report known or suspected child, elder (65 years of age or older), and dependent adult abuse according to the Penal Code Section 15630. The following are some guidelines to assist in dealing with these cases:

1. Identifying abuse:
   - Respondent provided information that gives the study staff reason to believe s/he has committed, or is currently engaging in, child/elder/dependent adult abuse.
   - Respondent is a minor/elder/dependent adult and has disclosed that s/he has been, or is currently being, physically/sexually abused.
   - Study staff witnessed or received information regarding unjustified or excessive physical punishment.
   - Child/elder/dependent adult has unexplained bruises or marks.
   - Child/elder/dependent adult presents signs of neglect.

## If study staff member believes the respondent may have committed, or may have been a victim of, physical and/or sexual abuse:

1. Assess the situation. If unsure about it, complete the interview if possible. But if the situation is volatile, stop the interview. Try to be as graceful and subtle as possible. For example, complete the form in progress and reschedule the rest of the interview. Study staff members should trust their sense of the situation, and act accordingly.
2. Seek consultation immediately. Report the information to the supervising counselor and determine if a report is warranted. In addition, the contact person within NPI for child/elder/dependent adult abuse cases is Carlyn Lampert, coordinator of the SCAN team (Suspected Child Abuse and Neglect). If advice/consultation is needed, page any of the SCAN team members by contacting the UCLA page operator at (310) 206-6301 and asking for the SCAN team (their page code is 95818). Ms. Lampert can also be contacted directly at (310) 825-0295.
3. If it is determined that a report should be made:

## Suspected Child Abuse:

1. Immediately complete the Department of Children and Family Services (DCFS) child abuse form (contained in Appendix I).
2. Call DCFS at (800) 540-4000 immediately to call in the report. Have the completed form at hand during the call, as the representative will need to obtain specific information about the incident. In addition, ask for the name of the DCFS worker on the call and document this name in the progress notes. *NOTE: STUDY STAFF’S RESPONSIBILTY IS TO ALLOW THE PROTECTIVE AGENCIES TO MAKE THE DECISION OF TAKING OR REFUSING THE REPORT. UCLA’S LIABILITY INSURANCE COVERS WHATEVER STUDY STAFF MIGHT DO IN RESPONSE TO THE SITUATION.*
3. Mail the completed form within 24 hours of the call (retain a copy of the form on site):

DCFS

3075 Wilshire Blvd., 5th Floor Los Angeles, CA 90010

## Suspected Elder/Dependent Adult Abuse:

1. Immediately complete the appropriate Adult Protective Services (APS) elder/dependent adult abuse form (contained in Appendix I).
2. If during business hours (Monday-Friday, 8:30am-5:00pm), fax the completed report immediately to (213) 738-6485. Retain the fax confirmation sheet with the report and document in the progress notes.
3. If after hours or on the weekend, immediately call in the report to Adult Protective Services at (800) 992-1660. Have the completed form at hand during the call, as the representative will need to get specific information about the incident. In addition, ask for the name of the APS worker on the call and document this name in the progress notes.
4. After the report has been faxed or called in, mail the completed form to: APS Central Intake

3333 Wilshire Blvd., 4th Floor Los Angeles, CA 90010

1. Call to follow-up on the status of the report at (213) 738-2669.
   - Remember, study staff is under no obligation to inform the participant that a report is being made.
2. Maintain contact with the study site supervisor throughout the process and document the details of the incident and reporting process thoroughly in the progress notes.

## Regulatory Affairs

The role of the Regulatory Affairs office during the conduct of a research study is to provide guidance on interpreting applicable regulations, as well as ensuring compliance with the applicable regulations.

## Protocol Violations

A Protocol Violation (PV) is defined as any violation or departure from the current IRB approved protocol by research personnel.

Examples of violations include but are not limited to the following:

- Consenting errors (e.g., research staff or participant forgets to sign or date, using an outdated version of the consent form)
- Randomization errors
- Unintentional deviations by research staff due to misunderstanding, oversight, or error
- Intentional deviations due to participant safety, treatment concerns, or for data purposes
- Incorrect administration of intervention(s) (e.g., forgetting a measure)

Participant non-compliance, such as failure to show for a scheduled visit, is NOT a protocol violation.

## Deviations from the Operations Manual that do not specifically violate the protocol will not be documented as protocol violations.

Repeated protocol violations may indicate the need for additional training of research staff, or for a protocol amendment if the PV is common. Protocol violations should be captured in the weekly progress notes as well as the Protocol Violations Log.
